# STATs Signaling Pathways in Acute Pancreatitis: Mechanisms and Regulation

**DOI:** 10.7150/ijms.126174

**Published:** 2026-03-30

**Authors:** Yang Peng, An-er Lin, Feng-ning Yang, Yan-min Tang, Jia-qi Yao, Yong Tang, Yi-fan Miao

**Affiliations:** 1 School of Acupuncture and Tuina, Chengdu University of Traditional Chinese Medicine, Chengdu, China.; 2 School of Clinical Medicine, Chengdu University of Traditional Chinese Medicine, Chengdu, China.; 3 Institute of Hepatopancreatobiliary Surgery, Chongqing General Hospital, Chongqing University, Chongqing 401147, China.; 4 International Collaborative Centre on Big Science Plan for Purinergic Signalling, Chengdu University of Traditional Chinese Medicine; School of Health and Rehabilitation, Chengdu University of Traditional Chinese Medicine, Chengdu, China.; 5 Department of Emergency Medicine, Hospital of Chengdu University of Traditional Chinese Medicine, Chengdu, China.

**Keywords:** Acute pancreatitis, The signal transducer and activator of transcription family, Traditional Chinese medicine, Pharmacological interventions

## Abstract

Acute pancreatitis (AP) is a potentially life-threatening inflammatory disorder of the pancreas that can progress from local injury to systemic inflammatory response and multi-organ dysfunction. Despite improvements in supportive treatment, there is still no effective drug therapy for AP. This highlights the necessity of clarifying its molecular mechanisms and exploring novel therapeutic strategies. Among the diverse signaling pathways implicated in the pathogenesis of AP, the significance of the signal transducer and activator of transcription (STAT) family has become increasingly evident. Building on the increasing recognition of the STATs family in AP, this review provides a systematic synthesis in three domains. The first addresses mechanistic studies of STATs-related signaling, the second focuses on pharmacological regulation with an emphasis on natural products and chemical drugs, and the third explores clinical evidence connecting STATs activity to severity and outcomes of AP. By synthesizing mechanistic, pharmacological, and clinical evidence, this review highlights the central role of STATs family members in AP. It underscores the therapeutic potential of natural medicines in targeting STATs pathways and suggests directions for future translational studies to develop effective interventions for AP.

## 1. Introduction

Acute pancreatitis (AP) is a common gastrointestinal emergency characterized by abrupt pancreatic inflammation and a highly variable clinical course 1. While most cases are mild and self-limiting, a considerable proportion progress rapidly to severe disease, accompanied by systemic inflammatory response syndrome (SIRS), multiple organ dysfunction syndrome (MODS), and even death. The global incidence of AP is rising, imposing a substantial clinical and socioeconomic burden 2. In AP, pancreatic acinar cell damage and local inflammatory activation serve as the starting point for systemic amplification, ultimately causing oxidative stress, mitochondrial injury, and disruption of intestinal and pulmonary barriers 3,4. Clarifying the mechanisms underlying this local-to-systemic progression is key to identifying effective therapeutic strategies.

Amidst the multiple signaling cascades involved in AP, accumulating evidence highlights the signal transducer and activator of transcription (STAT) family as a critical pathway driving disease progression. STATs proteins are cytoplasmic transcription factors that remain inactive under basal conditions and are rapidly activated in response to cytokines and growth factors 5,6. STATs activation is most commonly mediated via janus kinases (JAKs) in response to cytokines. Additionally, STATs can be activated via interleukin (IL)-6 or suppressor of cytokine signaling (SOCS) pathways. Although nuclear factor kappa-B (NF-κB) does not directly activate STATs, it can promote STATs activation indirectly by inducing upstream cytokines like IL-6 that engage the JAK/STAT axis 7. Once phosphorylated, STATs proteins form dimers, translocate into the nucleus, and control the expression of genes involved in immunity, inflammation, oxidative stress, and cell survival 8-10. The mammalian STATs family consists of seven major members with distinct biological functions. STAT1 and STAT2 coordinate antiviral defense and interferon signaling 11,12. STAT3 plays a key role in inflammation and tissue repair 13. STAT4, STAT5a and STAT5b regulate T-cell differentiation and hematopoiesis 14,15, while STAT6 contributes to Th2 immune responses and tissue remodeling 16. Aberrant activation of STATs signaling in AP acts as a central driver of disease progression by linking cytokine stimulation to pathological immune responses and systemic complications. However, the potential roles of other STATs members remain poorly characterized, as most existing studies have focused almost exclusively on STAT3.

**Figure 1:**
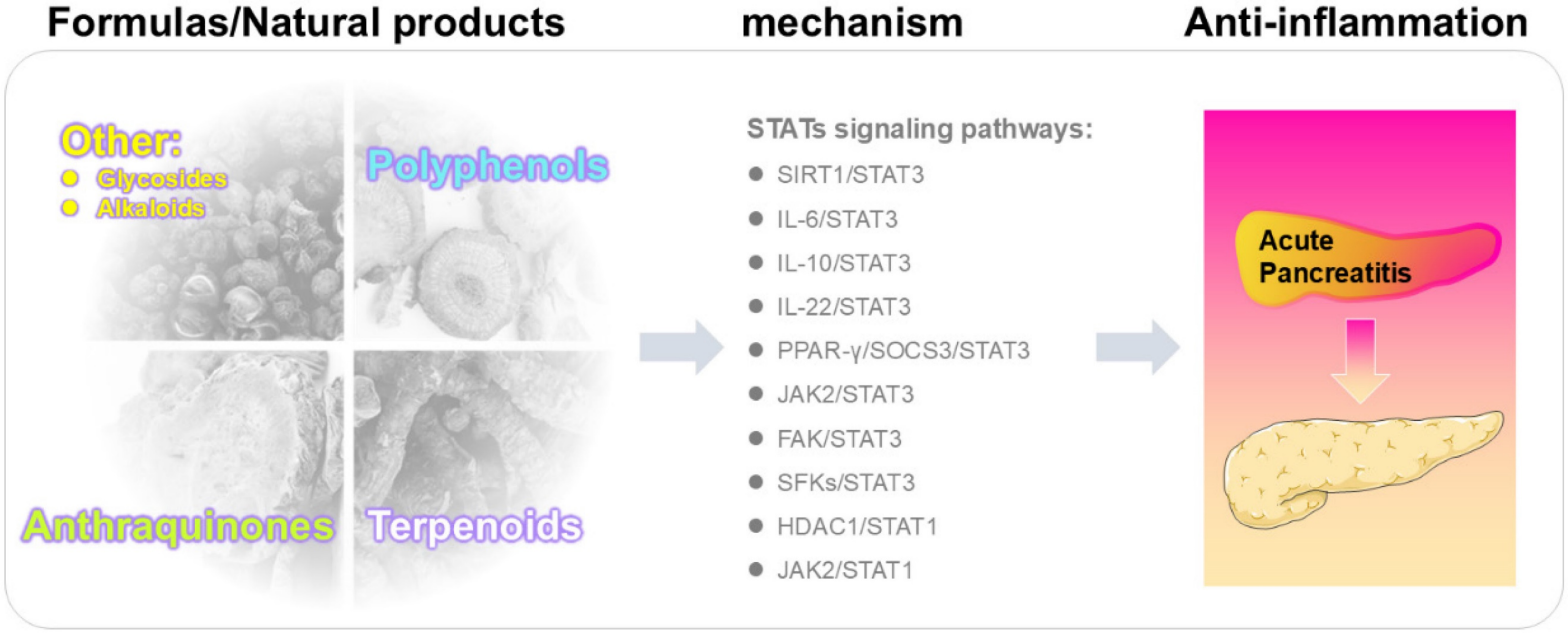
** Mechanism by which natural products alleviate acute pancreatitis**.

Several reviews have addressed JAK/STAT signaling in the context of AP and gastrointestinal diseases 17,18. Research to date has largely centered on STAT3 as the pivotal mediator of pathological signaling in AP, whereas STAT1 and other STATs family members have been only sporadically addressed, leaving their roles insufficiently defined. To address this gap, the present review provides the first comprehensive synthesis of the STATs family in AP. By providing a comprehensive and forward-looking analysis, this review not only highlights the mechanistic and therapeutic significance of the STATs family in AP but also offers a conceptual framework for the rational development of natural medicine-based and integrative therapeutic approaches (**Figure 1**).

## 2. Mechanistic Investigations

**Figure 2:**
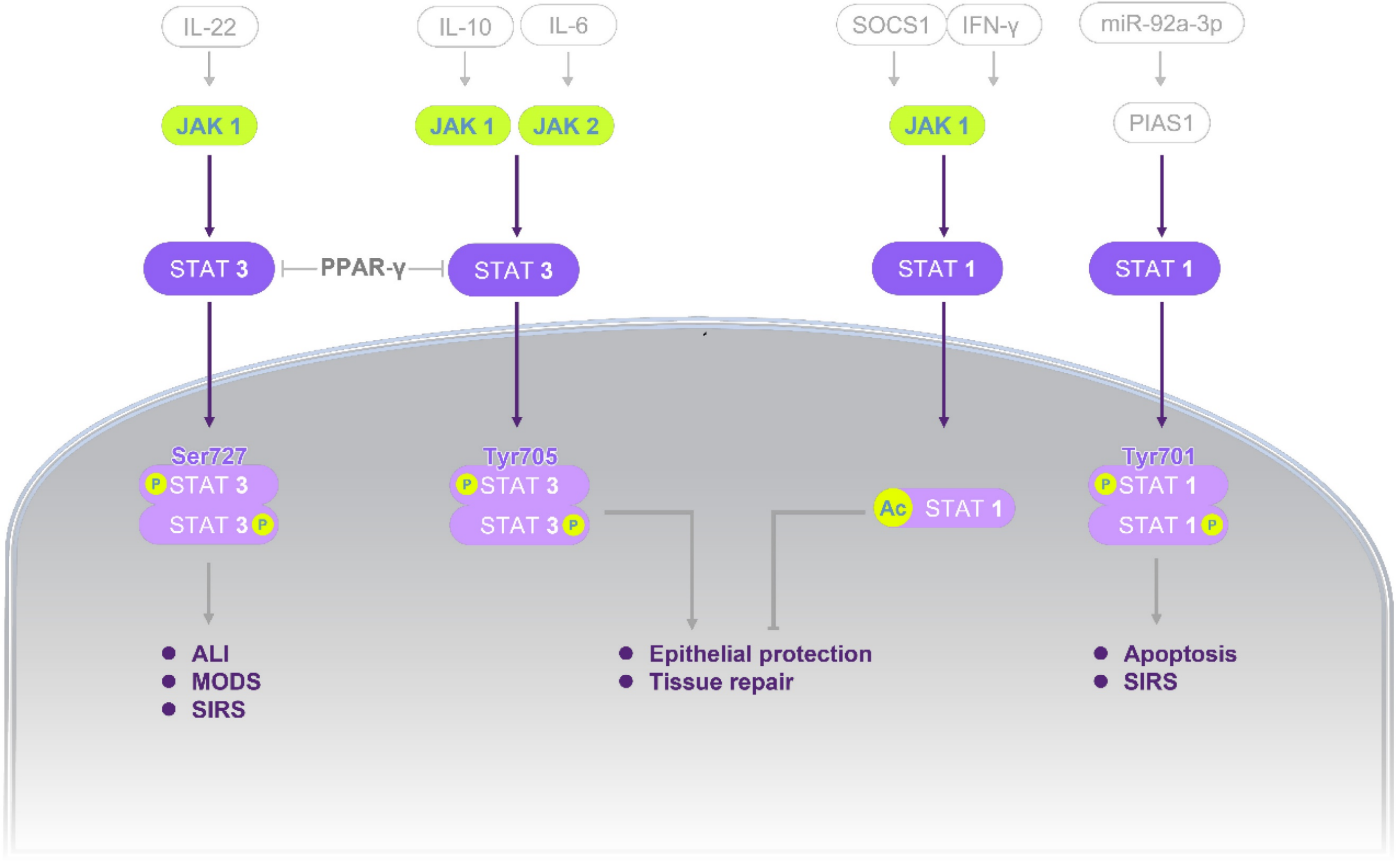
** STATs signaling governing injury-repair balance in AP.** In STAT3 axis (left), IL-22, IL-10, and IL-6 positively regulate JAK1 or JAK2 and thereby upregulate STAT3 activity. PPAR-γ functions as a connecting positive modulator across the parallel STAT3 branches. Intracellularly, p-STAT3 (Ser727) and p-STAT3 (Tyr705) were increased. Functionally, STAT3 activation enhances epithelial protection and tissue repair; in parallel, it upregulates chemokine production and inflammatory amplification, which increases macrophage and neutrophil recruitment and may progress to ALI and MODS. In STAT1 axis (right), IFN-γ activates JAK1/STAT1 pathway while SOCS1 negatively regulates this pathway. A second route involves PIAS1, which negatively regulates STAT1; miR-92a-3p downregulates PIAS1, then net-upregulating STAT1. Intracellular readouts show increased acetylation and phosphorylation of STAT1. Downstream, STAT1 activation upregulates NF-κB and COX-2 signaling and induces IL-17 as well as the pro-apoptotic mediators FasL, TRAIL, and Bax, culminating in SIRS and apoptosis. Standard arrows denote positive regulation (upregulation/activation) and T-shaped lines denote negative regulation (downregulation/inhibition). Abbreviations: Ac, acetylation; ALI, acute lung injury; AP, acute pancreatitis; Bax, Bcl-2-associated X protein; COX-2, cyclooxygenase-2; FasL, Fas ligand; IFN-γ, interferon-gamma; IL, Interleukin; JAK, janus kinase; miR, microRNA; MODS, multiple-organ dysfunction syndrome; NF-κB, nuclear factor kappa-B; P, phosphorylation; PIAS1, protein inhibitor of activated STAT1; PPAR-γ, peroxisome proliferator-activated receptor gamma; SIRS, systemic inflammatory response syndrome; SOCS1, suppressor of cytokine signaling 1; STAT, signal transducer and activator of transcription; TRAIL, tumor necrosis factor-related apoptosis-inducing ligand.

Mechanistic studies highlight the central role of STATs signaling in AP, integrating upstream inflammatory stimuli, downstream effector responses, and multiple pathological processes. A comprehensive overview of these mechanisms is summarized in **Figure 2** and **Tables 1-2**, providing the basis for subsequent discussion.

### 2.1 Activation of the STATs pathway: upstream signals and functional duality

#### 2.1.1. STAT1

Studies indicate that JAK1 acts as a critical upstream regulator of STAT1 signaling during the progression of AP. In cerulein (CER)-stimulated AR42J cells, the total and phosphorylated levels of JAK1, STAT1, and STAT3 increase synchronously with TNF-α, IL-1β, and IL-6 19. AG490 (tyrphostin AG490; tyrphostin B42) is a tyrosine kinase inhibitor that targets EGFR, STAT3, and JAK2/3. Treatment with AG490 significantly suppresses these inflammatory responses, suggesting therapeutic potential during the hyperacute phase 19. Furthermore, the JAK2/STAT1 pathway contributes to disease progression, as demonstrated by the effective inhibition of its activation through AG490 or FKN-siRNA 20. Notably, despite being a canonical STAT1 activator, interferon gamma (IFN-γ) may exert context-dependent protective effects in CER-induced AP by promoting STAT1 acetylation, thereby inhibiting NF-κB nuclear translocation and downstream pro-inflammatory signaling 21. Additionally, the miR-155/SOCS1/STAT1 axis is involved in immune dysregulation, where miR-155 inhibition restores SOCS1-mediated negative feedback on STAT1 signaling and reduces IL-17-driven injury 22.

#### 2.1.2. STAT3

JAK2-mediated STAT3 phosphorylation is essential for local and systemic inflammation. The activation of the JAK2/STAT3 pathway promotes macrophage polarization, whereas pharmacological inhibition using AG490 effectively reduces the levels of p-JAK2 and p-STAT3 at the Ser727 residue 23. FXYD domain-containing protein 5 (FXYD5) and NADPH oxidase act as crucial upstream triggers, linking transmembrane signaling and oxidative stress to the JAK2/STAT3 pathway 24,25. Beyond these molecular triggers, activation often varies by etiology. Bile acids in biliary AP models induce rapid JAK2/STAT3 activation linked to severe oxidative stress 26, whereas in alcoholic AP, ethanol metabolites and reactive oxygen species may activate STAT3 as a protective response to resolve cellular redox imbalance 27. Beyond JAK2-dependent mechanisms, peroxisome proliferator-activated receptor gamma (PPAR-γ) inhibits STAT3 through direct interaction 28, while the neurokinin 1 receptor and Src family kinases upregulate its expression 29. Interestingly, in biliary models, oxidized phospholipids can exert dual effects by triggering STAT3-mediated inflammation at low concentrations or recruiting PPAR-γ to inhibit such signaling at higher levels 30.

IL-6 remains a primary upstream activator that recruits JAK2 via the gp130 receptor complex to upregulate p-STAT3 at the Tyr705 site 31. The intensity of this signal is significantly altered by metabolic factors such as obesity, which leads to prolonged elevations of IL-6 and sustained STAT3 phosphorylation that differs from the transient activation seen in lean models 32. Within the first three hours after AP induction, IL-6 expression is typically upregulated, and its neutralization has been shown to reduce p-STAT3 levels while alleviating associated lung injury 33. The STAT3 signalosome is further balanced by non-coding RNAs and counter-regulatory cytokines. While miR-148a and miR-551b-5p provide opposing regulatory frameworks 34,35, IL-10 and IL-22 further define the functional duality of STAT3. Specifically, IL-10-driven STAT3 activation can, under certain viral contexts, paradoxically amplify inflammation 36, whereas IL-22-mediated signaling contributes to barrier repair and cellular protection 37. Notably, the protective IL-22/STAT3 response in the colon tends to decline more rapidly than pro-inflammatory markers, which potentially increases the risk of intestinal barrier failure as AP progresses 38.

Collectively, the net biological output of STAT3 in AP reflects a stimulus and cell type specific integration of competing upstream signals. The IL-6/STAT3 axis primarily acts as a pro-inflammatory driver in acinar cells and recruited neutrophils, where it contributes to the escalation of local injury into a systemic cytokine storm 31,33. In contrast, IL-22-mediated STAT3 signaling functions as a protective pathway in acinar and intestinal epithelial cells, playing a role in maintaining barrier integrity and promoting tissue repair 37,38. Furthermore, while IL-10 is traditionally recognized as a potent anti-inflammatory cytokine, its role via STAT3 can be paradoxically pro-inflammatory depending on the pathological context, such as in certain viral models 36. These multifaceted roles identify STAT3 as a key regulator that balances detrimental inflammatory responses with beneficial tissue repair based on the molecular stimulus and cellular environment.

#### 2.1.3. Dynamic activation patterns of STATs across disease stages

STAT activation evolves dynamically. In the hyperacute phase, TNF-α enhances the nuclear binding of STAT1 and STAT3 within 30 minutes 39. Simultaneously, hydrostatic pressure can directly enhance NF-κB p65 and STAT3 signaling, thereby inducing inflammation, which suggests a critical regulatory role of STAT3 in sensing mechanical stress within a 24 hour experimental timeframe 40. Subsequently, in pressure-induced or biliary pancreatitis, IL-6 trans-signaling drives pathogenic responses via dual STAT3 phosphorylation (Tyr705/Ser727) 31,41. While Tyr705 phosphorylation is essential for STAT3 dimerization, the phosphorylation at Ser727 is particularly critical for enhancing transcriptional efficacy and maintaining mitochondrial bioenergetics, which may help preserve acinar cell integrity during early injury 42,43. This dual activation also induces C-X-C motif chemokine ligand 1 and promotes neutrophil recruitment 31. In viral AP, STAT3 and STAT1 exhibit an antagonistic relationship where excessive STAT3 activation might impair viral clearance 44. Temporally, while moderate STAT3 activation promotes autophagic flux and alleviate edema, sustained activation drives chronic fibrosis and multi-organ injury 45.

### 2.2 Downstream effectors of STATs signaling

STAT3 executes a broad transcriptional program constrained by intrinsic feedback. Inhibition of the JAK2/STAT3 pathway typically downregulates pro-inflammatory cytokines, including IL-6, IL-1β, and TNF-α, mitigating SAP severity and systemic injury 46,47. The IL-6/STAT3 signaling axis serves as a critical upstream pathway initiating these downstream inflammatory responses, directly promoting the expression of pro-inflammatory cytokines such as TNF-α 48,49. Simultaneously, this axis forms a STAT3/SOCS3 negative feedback module 50. The downstream consequences of STAT3 activation are notably stage-dependent. In the acute phase, the neutralization of IL-6 may increase cellular apoptosis to mitigate SAP severity 33. However, as the disease enters the resolution phase, p-STAT3 activation appears to trigger the acinar-to-ductal metaplasia (ADM) process, which is increasingly recognized as a self-protective mechanism that reduces acinar necrosis and initiates tissue remodeling 51. Mechanistically, STAT3 is also considered indispensable for the production of pancreatitis-associated protein 1 (PAP1), which is a key protein that suppresses necrosis and facilitates pancreatic repair 52.

Furthermore, the STAT3-dependent chemokine network links acinar signaling to immune cell recruitment, and its persistent activation in the chronic phase correlates with tissue fibrosis 53. In obesity-related contexts, this sustained activation may even favor a protumorigenic microenvironment through the induction of matrix metalloproteinase-7 32. To maintain homeostasis, SOCS3 provides self-limiting negative feedback to control inflammation. Specifically, PAP/hepatocarcinoma-intestine-pancreas (HIP) proteins induce STAT3-mediated SOCS3 transcription, which subsequently inhibits JAK phosphorylation and NF-κB activation 54,55. Similarly, sirtuin 1 (SIRT1)-mediated STAT3 deacetylation reduces its transcriptional activity and decreases inflammatory cytokine expression, indicating that SIRT1 helps mitigate AP by inactivating STAT3 56.

### 2.3 Pathological mechanisms mediated by STATs signaling

During the progression of AP, early local inflammation can rapidly escalate into a SIRS, which is frequently characterized by a cytokine storm. STAT family members, particularly STAT1 and STAT3, are involved in orchestrating these inflammatory circuits by regulating various pro-inflammatory cytokines, chemokines, and immune cell functions. STAT3 appears to promote inflammation via the activation of the IL-6/STAT3 pathway 33 or the IL-10/STAT3 axis 36, which is particularly relevant in viral AP. This process includes the induction of chemokines 56,57 and a potential synergistic interaction with NF-κB 58, which collectively enhance neutrophil and macrophage infiltration 59 and exacerbate pancreatic injury 60,61. STAT1 similarly amplifies inflammation through transcriptional coordination with NF-κB 62,63. However, in addition to its pro-inflammatory role, STAT3 exerts protective effects in AP through multiple mechanisms, including the induction of anti-inflammatory PAP1 52, and mediation of protection related to T-cell protein tyrosine phosphatase deficiency 64.

Beyond inflammation, STATs signaling drives parenchymal damage. In acinar cells, TNF-α-induced STAT1/STAT3 activation leads to premature digestive enzyme secretion such as amylase and trypsinogen 39. The IL-6/JAK2/STAT3 axis is suggested to be a relevant node for acinar injury, as the inhibition of this pathway has been shown to reduce serum levels of amylase and lipase while alleviating tissue edema 26,65. STAT3 also mediates lipotoxic signals and may promote ferroptosis via the CLDN4/JAK2/STAT3 axis 66,67. Furthermore, the temporal control of STAT3 signaling is likely important for tissue homeostasis because uncontrolled signaling, which can result from IL-22BP deficiency, is associated with persistent ADM and delayed tissue recovery 45. Conversely, site-specific phosphorylation (Tyr705/Ser727) via the FAK/STAT3 axis preserves mitochondrial fitness and limit apoptotic demise 42,43. This parenchymal damage extends to endocrine islets, where the IFN-γ/STAT1 axis drives β-cell dysfunction and reduced insulin biosynthesis 68,69. Although basal STAT3 preserves β-cell integrity 70, inflammatory STAT3 hyperactivation impairs insulin secretion 71,72.

As the disease progresses toward its severe form, STAT signaling facilitates the dissemination of pathological signals to extra-pancreatic organs, leading to systemic complications and chronic sequelae. During the transition to severe AP, extra-pancreatic organs are often among the earliest affected 73,74. The IL-22/STAT3 axis potentially facilitates the shift from local injury to systemic complications by regulating epithelial autophagy and immune responses 45. The IL-6/STAT3 pathway is closely linked to multi-organ fibrosis, potentially through fibroblast activation that drives structural remodeling of the kidney, lung, and vasculature 75-77. Abnormal JAK/STAT3 activation via ADAM17-mediated IL-6 trans-signaling facilitates the spread of pathological signals beyond the pancreas 75,77. Recombinant IL-22 and runt-associated transcription factor 3 have been suggested to mitigate pulmonary injury through mechanisms involving STAT3 signaling 47,78. Similarly, the IL-22/STAT3 and IL-10/STAT3 axes are thought to contribute to the preservation of the intestinal barrier and blood-brain barrier integrity 38,79,80. Furthermore, in the context of obesity-related AP, IL-6 may prolong the inflammatory response and potentially promote a tumorigenic microenvironment through sustained STAT3 activation 32. Essentially, this indicates that when STAT3 remains active, the initial acute damage transitions into a long-term problem that reshapes the pancreatic tissue, making the local environment more vulnerable to cancer development. However, despite the extensive documentation of the STAT1/3 axes, the roles of other family members in pancreatic pathology are often overlooked. For instance, the involvement of lesser-studied members such as STAT2 and STAT4 in coordinating the Th1/Th17 response during the acute-to-chronic transition remains a critical knowledge gap. Moreover, while acinar cell injury is well-characterized, how STAT signaling, particularly underdeveloped members such as STAT5, regulates ductal bicarbonate secretion to mitigate protease-driven injury is yet to be elucidated. Bridging these gaps in isoform-specific signaling across different cell types is essential for a holistic understanding of STAT-mediated regulation in AP.

## 3. Pharmacological studies

**Figure 3:**
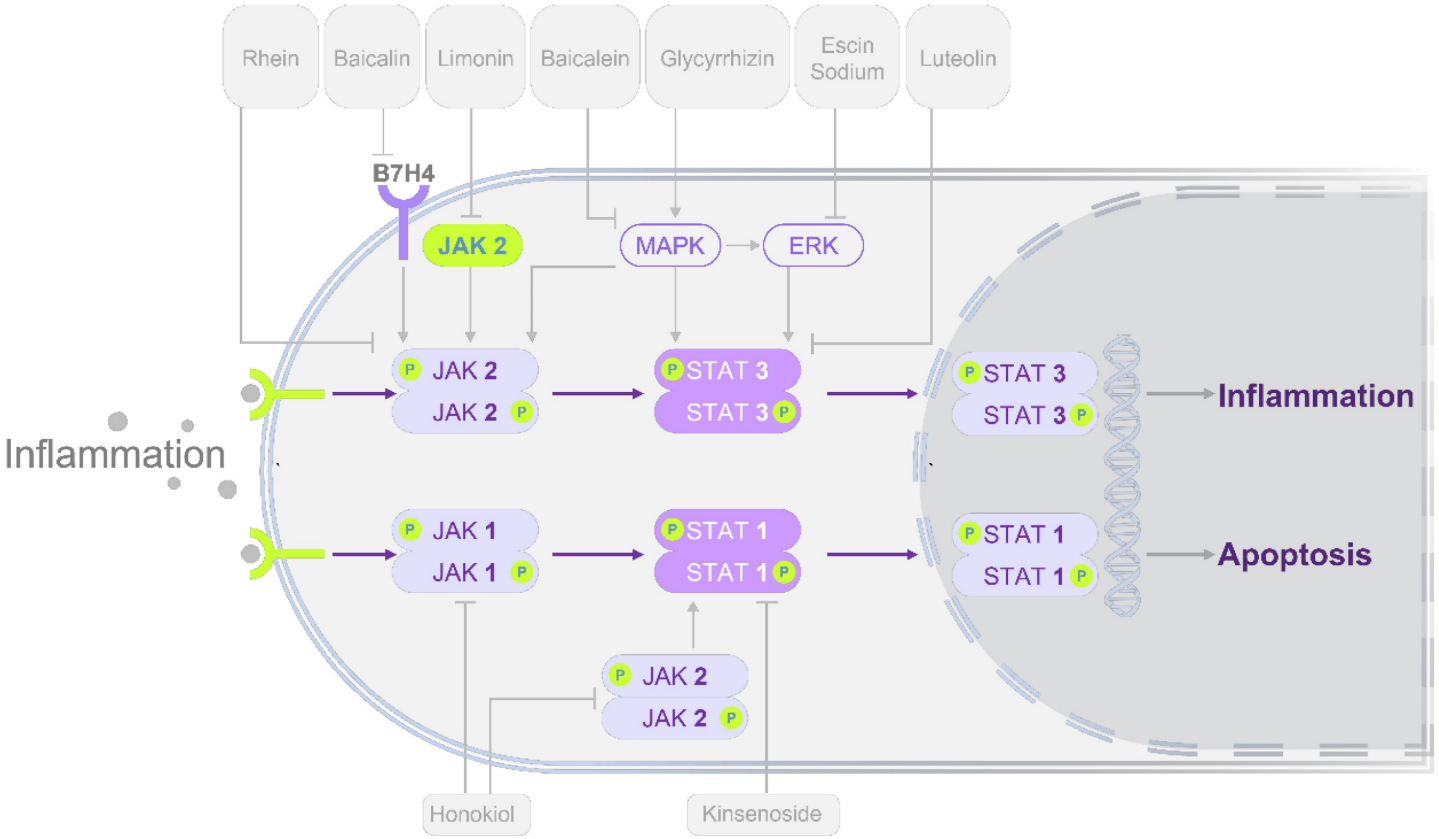
** Modulation of STATs signaling pathways by herbal-derived single compounds in alleviating AP.** Most compounds exert protective effects primarily through inhibition of the STAT3 pathway, while a subset acts via STAT1 modulation. In terms of STAT3 regulation, the majority of monomers act through the JAK2/STAT3 or MAPK/ERK/STAT3 pathways. Rhein reduces pancreatic injury by directly inhibiting phosphorylation of molecules in the JAK2/STAT3 pathway. Baicalein and baicalin act by suppressing upstream regulators B7H4 and MAPK, thereby modulating the JAK2/STAT3 cascade. In contrast, glycyrrhizin alleviates AP by inhibiting phosphorylation along the MAPK/ERK/STAT3/AKT axis. Similarly, escin sodium exerts protective effects by suppressing ERK/STAT3 pathway. Direct STAT3-targeting compounds include luteolin, daphnetin, and colchicine. At the STAT1 level, honokiol reduces AP by inhibiting JAK/STAT1 phosphorylation, while kinsenoside protects against AP by binding TLR4 and downregulating p-STAT1. These regulatory mechanisms provide potential therapeutic targets for AP. Standard arrows denote positive regulation (upregulation/activation) and T-shaped lines denote negative regulation (downregulation/inhibition). Abbreviations: AKT, protein kinase B; AP, acute pancreatitis; B7H4, B7 homolog 4; ERK, extracellular signal-regulated kinase; JAK, janus kinase; MAPK, mitogen-activated protein kinase; PPAR-γ, peroxisome proliferator-activated receptor gamma; Ser, serine; SOCS3, suppressor of cytokine signaling 3; STAT, signal transducer and activator of transcription; TLR4, toll-like receptor 4.

Building on mechanistic insights, pharmacological studies have demonstrated that modulation of STATs signaling can alleviate pancreatic injury and systemic complications. These interventions span multiple categories, including multi-component herbal formulas, bioactive natural compounds, and chemical agents, each with distinct regulatory effects on STATs activity. An overview of these pharmacological findings is presented in **Figure 3** and **Tables 3-4**, which highlight both shared and unique therapeutic mechanisms.

### 3.1 Herbal formulas

Da Cheng Qi Decoction (DCQD), a classical formula recorded in Shang Han Lun, consists of *Rheum palmatum* L. (Da Huang), *Magnolia officinalis* Rehd. et Wils. (Hou Po), *Citrus aurantium* L. (Zhi Shi), and *Natrii Sulfas* (Mang Xiao). Experimental studies have demonstrated that DCQD markedly reduces the protein expression of p-JAK2 (Tyr1007/1008) and p-STAT3 (Tyr705) *in vitro*, with an effect comparable to that of specific JAK2 or STAT3 inhibitors, suggesting that its therapeutic action primarily involves antagonism of the JAK2/STAT3 pathway 65. High-performance liquid chromatography (HPLC) identified nine major active components in DCQD, among which rhein has been confirmed to confer protection against AP through modulation of the STAT3 signaling pathway 65,81,82. Moreover, DCQD-mediated inhibition of JAK2/STAT3 signaling also attenuated SAP-associated intestinal injury *in vivo* 82.

Chaiqin Chengqi Decoction (CQCQD) and Qingyi Granules (QYG), both derived from the combination of DCQD and Dachaihu Decoction, have been shown to modulate STAT3-mediated signaling in AP 83,84. CQCQD suppresses p-JAK2 and p-STAT3 expression *in vitro* and *in vivo*, thereby ameliorating CER-induced pancreatic injury via inhibition of the JAK2/STAT3 pathway 83. Early administration of QYG in AP patients with acute respiratory distress syndrome significantly reduced hospital stay and mortality 84. Consistent with these clinical findings, QYG was also reported to inhibit the IL-6/STAT3 signaling axis in a sodium taurocholate (NaT)-induced SAP rat model, mitigating both pancreatic and pulmonary injury 84. Additional related formulations, including Chaihuang Qingyi Huoxue Granule and Qingyi Decoction, similarly attenuate AP progression through downregulation of STAT3 signaling, primarily involving the phosphoinositide 3-kinase /protein kinase B (PI3K/AKT) and NF-κB/nucleotide-binding oligomerization domain like receptor (NLR) family pyrin domain containing 3 (NLRP3) pathways 85,86.

Da-Huang-Fu-Zi-Tang (DHFZT), a classical formula originally described in Synopsis of Golden Chamber (Jin Kui Yao Lue), is composed of *Rheum palmatum* L. (Da Huang), *Aconitum carmichaelii* Debx. (Fu Zi), *Asarum heterotropoides* F. Schmidt (Xi Xin). Previous studies have shown that DHFZT alleviates inflammatory responses, promotes intestinal motility, and exerts protective effects in SAP 87. A multicenter randomized controlled trial further demonstrated that DHFZT effectively reduced hospital stay, decreased mortality, and improved overall clinical outcomes in SAP patients 88. Mechanistic investigations in a NaT-induced SAP rat model revealed that DHFZT significantly suppresses inflammatory factor expression and inhibits the activation of p-JAK2 and p-STAT3, suggesting that its therapeutic effects involve antagonism of the JAK2/STAT3 signaling pathway, thereby mitigating AP progression and alleviating SAP-associated liver injury 87.

Shenmai injection (SMI), a sterile preparation derived from *Panax ginseng* C.A. Mey. (Ren Shen) and *Ophiopogon japonicus* (L.f.) Ker Gawl. (Mai Dong), contains four major active components identified by ultra-high-performance liquid chromatography-mass spectrometry: ginsenoside Rb1, ginsenoside Rg1, ginsenoside Re, and ophiopogonin D 89. *In vitro* studies indicated that these compounds enhance the viability of 266-6 cells 89. Further investigations in both cell-based and animal models of AP demonstrated that SMI confers protective effects by inhibiting the IL-6/STAT3 signaling pathway, thereby attenuating pancreatic injury and associated inflammatory responses 89.

*Rheum palmatum L.* and *Salvia miltiorrhiza Bge.* (Dahuang and Danshen, DH-DS), recorded in Shennong's Classic of Materia Medica (Shen Nong Ben Cao Jing) for their properties of clearing dampness-heat and resolving stagnation, have been investigated for their potential to modulate the pathogenesis of AP. Experimental studies demonstrated that DH-DS significantly reduces inflammatory cytokine levels and suppresses the expression of p-JAK2, p-STAT3, and IL-17A in the CER-induced AP model. These effects indicate that DH-DS exerts protective actions by modulating the JAK2/STAT3/IL-17A signaling axis 90. Mechanistically, upregulation of IL-17A promotes Th17 cell differentiation, thereby amplifying inflammatory responses. In addition, molecular docking and dynamics simulations confirmed a strong binding affinity between DH-DS, particularly its active component Danshexinkum D, and the STAT3 protein, supporting the molecular basis for its regulatory effects on the STAT3 pathway 90.

Traditional chinese medicine (TCM) formulations exert multi-component, multi-target regulatory effects in the treatment of AP. Studies have demonstrated that commonly used formulas, including DCQD, CQCQD, QYG, and DHFZT, can downregulate inflammatory cytokines, inhibit the STAT3 signaling pathway, reduce acinar cell apoptosis, and protect against AP-associated intestinal and pulmonary injuries. Mechanistic studies have further identified active components of DCQD, QYG, DHFZT, and SMI via HPLC analysis, and research on DCQD and CQCQD has employed specific JAK2/STAT3 inhibitors to provide pharmacological validation of their effects. Despite these advances, several limitations remain. First, the experimental models employed are relatively homogeneous, and investigations have largely focused on STAT3, with limited systematic exploration of other STATs family members, restricting a comprehensive understanding of the STATs network in AP. Second, genetic editing approaches, such as CRISPR-Cas9, have not been applied to directly validate putative targets, limiting mechanistic depth. Third, chemical profiling does not equate to mechanistic verification. Although most formulas undergo HPLC analysis to identify constituents, the functional link between these specific compounds and STAT3 inhibition is rarely established experimentally. Their relevance is often merely predicted via network pharmacology or molecular docking, without direct evidence delineating which components actually drive the pathway modulation. Consequently, the active principles remain largely speculative rather than validated, representing a critical gap between phytochemical characterization and mechanistic proof. Moreover, clinical safety profiles and potential adverse effects of these formulations remain inadequately characterized.

### 3.2 Herbal-derived single compounds

#### 3.2.1. Polyphenols

Polyphenolic monomers, which are abundant in TCM herbs such as *Scutellaria baicalensis* Georgi (Huang Qin) and *Magnolia officinalis* Rehd. et Wils. (Hou Po), mainly comprise flavonoids and phenylpropanoids. Among flavonoid monomers, those shown to modulate the STATs signaling pathway in AP include baicalein (5,6,7-trihydroxyflavone), baicalin (7-D-glucuronic acid-5,6-dihydroxyflavone), and luteolin (3',4',5,7-tetrahydroxyflavone). Similarly, phenylpropanoid monomers with reported STATs-regulatory activity include daphnetin (7,8-dihydroxy-2H-1-benzopyran-2-one) and honokiol (3',5-di(prop-2-en-1-yl)[1,1'-biphenyl]-2,4'-diol). Moreover, curcumin (1,6-Heptadiene-3,5-dione, 1,7-bis(4-hydroxy-3-methoxyphenyl)-, (E,E)-), 4-(2-acetoxy-3-((R)-3-(benzylthio)-1-methoxy-1-oxopropan-2-ylamino)-3-oxopropyl)-1,2-phenylene diacetate (DSC), a danshensu-derived compound and (R)-4,6-dimethoxy-3-(4-methoxy phenyl)-2,3-dihydro-1H-indanone [(R)-TML104] have also been shown to possess relevant regulatory effects on the STATs pathway. Current studies indicate that most monomeric compounds confer protective effects against AP primarily via modulation of the STAT3 signaling pathway. Honokiol is notable as the only compound reported to influence the STAT1 pathway. Baicalein and baicalin have been shown to downregulate the protein expression of p-JAK2 and p-STAT3, thereby inhibiting JAK2/STAT3 signaling and attenuating AP-related injury 91,92. Interestingly, their upstream mechanisms differ: baicalein regulates mitogen-activated protein kinase (MAPK) activity 91, whereas baicalin modulates the expression of B7 homolog 4, an established biomarker of type I diabetes progression 92. Notably, luteolin downregulates p-STAT3 (Tyr705), the inflammatory transcription factor SRY-box 9, and phosphorylated epidermal growth factor receptor, thereby suppressing ADM, reversing epithelial-mesenchymal transition, and blocking the progression from pancreatitis to pancreatic cancer 93. Several studies have investigated how monomeric compounds protect extra-pancreatic organs in AP. For example, daphnetin downregulated the expression of p-JAK2 (Tyr1007) and p-STAT3 (Tyr705) in lung tissue, thereby attenuating pancreatic and pulmonary injury in an arginine (ARG)-induced AP model 94. Honokiol suppressed high-mobility group protein B1 (HMGB1), p-JAK1 (Tyr1034/1035), p-JAK2 (Tyr1007/1008), and p-STAT1 (Ser727) protein levels, leading to inhibition of the JAK1/2-STAT1 pathway and amelioration of SAP-associated intestinal injury 95. Under inflammatory conditions, macrophages release substantial amounts of HMGB1 into the intestinal tract, thereby compromising gut barrier integrity and exacerbating SAP. Similarly, butyrate, a short-chain fatty acid, alleviates intestinal inflammation by inhibiting the GPR109A-NLRP3 axis and suppressing the histone deacetylase 1 (HDAC1)/STAT1/NLRP3 pathway in the pancreas, resulting in reduced pancreatic and intestinal damage in the CER-AP model 96. Additionally, curcumin downregulated renal p-JAK2 and p-STAT3 expression, thereby mitigating pancreatic and renal injury in a NaT-AP model 97. Moreover, both *in vivo* and *in vitro* models of CER-induced AP have demonstrated that DSC downregulates p-STAT3 (Tyr705) and the NLRP3 inflammasome, thereby inhibiting the STAT3/NLRP3 signaling axis and modulating macrophage polarization, which ultimately attenuates pancreatic injury 98. As another novel compound, (R)-TML104 has been shown in CER-induced AP to markedly enhance the interaction between SIRT1 and STAT3, leading to reduced STAT3 acetylation. Consequently, it attenuates the IL-6/STAT3 pathway mediated inflammatory response, decreases chemokine expression, modulates the immune microenvironment, and ultimately mitigates pancreatic injury 56. In addition, sodium selenite, commonly supplemented in AP patients with selenium deficiency, has been shown to suppress the MAPK/NF-κB/STAT3 pathway, conferring protection against AP 99. Overall, polyphenolic monomers modulate STATs signaling through distinct patterns: baicalein, baicalin, daphnetin and curcumin downregulate both p-JAK2 and p-STAT3; luteolin, DSC and (R)-TML104 reduce p-STAT3 levels without documented JAK2 involvement; honokiol uniquely inhibits the JAK1/2-STAT1 axis. However, a critical gap remains across this class of compounds: direct evidence of physical interaction with STAT proteins is largely absent. To date, molecular docking predictions or biophysical binding assays are missing for the majority of these polyphenols. While functional causality has been supported by inhibitor-based studies, genetic knockout models, or protein interaction assays for compounds such as honokiol, butyrate, and (R)-TML104, the link between the remaining monomers and STAT3 inhibition remains primarily correlative. Consequently, future research requires definitive validation using genetic loss-of-function models to confirm these targets.

#### 3.2.2. Terpenoids

Terpenoids monomers, commonly found in traditional medicinal herbs, have been shown to exert anti-inflammatory effects in AP primarily by modulating the STAT3 signaling pathway. Studies indicate that picroside II (6-vanilloylcatalpol) and lutein suppress the phosphorylation of JAK2 and STAT3, while limonin (7,16-dioxo-7,16-dideoxylimondiol) reduces JAK2 and p-STAT3 activation 100-102. These effects converge on inhibition of the JAK2/STAT3 pathway, thereby ameliorating AP. Specifically, picroside II 100 and limonin 101 directly suppress the activation of this pathway, whereas lutein 102 indirectly modulates its activity by upregulating PPAR-γ and SOCS3. In addition, escin sodium and glycyrrhizin attenuate pancreatic injury by reducing p-extracellular signal-regulated kinase (ERK)1/2 and p-STAT3 levels, thereby inhibiting the ERK/STAT3 pathway 103,104. Notably, computational docking and molecular dynamics simulations suggest that glycyrrhizin potentially binds to MAPK3 to form a stable complex, thereby implying a suppressive effect on the MAPK/ERK/STAT3/AKT signaling axis and a subsequent reduction of necrosis in pancreatic acinar cells (PACs) 104. Notably, glycyrrhizin is the only terpenoid in this group with a proposed binding mechanism. However, this interaction remains *in silico* and awaits physical validation. For all other terpenoids, the molecular basis of STAT3 inhibition remains entirely undefined. Without any target engagement data—even at the predictive level—their suppressive effects on STAT3 signaling are inferred solely from observed changes in phosphorylation, which should be considered correlative rather than causative.

#### 3.2.3. Anthraquinones

Rhein (4,5-dihydroxyanthraquinone-2-carboxylic acid), a major active component of DCQD, has been shown to exert notable anti-inflammatory effects 105. Previous studies have demonstrated that rhein alleviates CER plus lipopolysaccharide-induced SAP by modulating glycerophospholipid metabolism in pancreatic tissue 106. Furthermore, recent studies consistently indicate that rhein downregulates the expression of p-JAK2 (Tyr1007/1008) and p-STAT3 (Tyr705), thereby suppressing the JAK2/STAT3 signaling pathway and attenuating AP 81. Notably, molecular docking studies suggest that rhein potentially binds to JAK2 with binding sites that partially overlap the ATP pocket, which provides a predictive structural basis for its inhibitory role 81. However, this interaction awaits biophysical validation to confirm actual target engagement. Moreover, the specific quantitative contribution of rhein to the overall efficacy of the DCQD formula remains undefined, and its clinical translation for AP treatment has yet to be initiated.

#### 3.2.4. Others

Alkaloid and glycoside constituents similarly modulate inflammation in AP through regulation of the STATs signaling pathway. Representative compounds include the alkaloids colchicine (N-Acetyl trimethylcolchicinic acid methylether) and rutaecarpine (8,13-dihydroindolo(2',3':3,4)pyrido(2,1-b)quinazolin-5(7H)-one), along with the glycoside kinsenoside ((3R)-5-oxooxolan-3-yl beta-D-glucopyranoside). Colchicine has been shown to inhibit p-STAT3 expression in pancreatic and lung tissues, reduce inflammatory cytokine levels, and attenuate oxidative stress and apoptosis, thereby mitigating pancreatic and pulmonary injury in a NaT-induced SAP rat model 107. Likewise, rutaecarpine downregulates p-STAT3 expression and suppresses associated inflammatory pathways, including MAPK and NF-κB, thereby exerting protective effects against AP 108. Distinct from the previously discussed monomers, both *in vivo* and *in vitro* studies have shown that kinsenoside downregulates p-STAT1 and reduces inflammatory cytokine levels, inhibits the toll-like receptor 4 (TLR4)-STAT1 pathway, and modulates M1 macrophage polarization, thereby attenuating pancreatic and pulmonary injury in a SAP mouse model and decreasing apoptosis in CER-treated PACs 109. Network pharmacology analysis identified TLR4 as a key target mediating kinsenoside's protective effects in AP, a conclusion further supported by molecular docking results showing strong binding affinity between kinsenoside and TLR4 109. Similarly, lactate (2-hydroxypropanoate), a glycolytic end-product, promotes the shift of macrophages from the M1 to M2 phenotype, suppresses the GPR132 (a macrophage membrane receptor)/JAK2/STAT1 signaling axis, reduces inflammatory cytokine secretion, and improves the immune microenvironment, ultimately conferring protection against AP 110. Colchicine and rutaecarpine further expand the range of potential STAT3 inhibitors, while kinsenoside and lactate appear to shift the regulatory focus toward STAT1 through suggested upstream targets such as TLR4 and GPR132 107-110. However, the current evidentiary basis for these compounds remains preliminary, as it has not yet been demonstrated that any of these agents directly interact with components of the STATs signaling pathways. Furthermore, because no inhibitor-based experiments or genetic loss-of-function studies have been reported, the functional causality between these compounds and their observed effects remains largely untested. The STAT1-targeting candidates, despite findings from molecular docking or pharmacological inferences, currently lack biophysical validation and causal confirmation, which represents a research gap similar to the limitations previously observed for STAT3-oriented monomers.

Monomeric compounds exert multi-target regulatory effects on inflammatory signaling pathways in AP. Current studies indicate that a majority of investigated monomers alleviate AP-associated inflammation by downregulating JAK2/STAT3 pathway activity, including inhibition of JAK2 and STAT3 phosphorylation and reduction of STAT3 nuclear translocation. Notably, some monomers confer protection against extra-pancreatic organ injury. Daphnetin, colchicine, and kinsenoside ameliorate AP-associated lung damage via STATs signaling inhibition, curcumin mitigates AP-related renal injury through suppression of the JAK2/STAT3 pathway, honokiol and butyrate alleviate intestinal injury via inhibition of STAT1 signaling. Particularly, luteolin has revealed a potential role for STAT3 in the transition from AP inflammation to carcinogenesis. Furthermore, DSC, kinsenoside, and lactate promote M1-to-M2 macrophage polarization via STATs pathway modulation, thereby regulating the immune microenvironment and mitigating AP progression. Nevertheless, current studies are largely constrained by the reliance on single animal models and the absence of dynamic mechanistic analyses across multiple models and time points. Consistent with research on TCM formulas, studies on monomeric compounds have predominantly focused on the STAT3 pathway, with only a few, including honokiol, butyrate, and kinsenoside, examining STAT1 regulation. Systematic investigations into other STATs family members remain limited. Mechanistically, few studies have employed pharmacological interventions using small-molecule inhibitors or agonists, and genetic editing approaches have rarely been applied to functionally validate specific STATs pathway targets. Furthermore, most experimental studies tend to utilize prophylactic dosing regimens, which frequently deviate from the actual clinical therapeutic window where treatment usually commences only after the onset of systemic inflammation. This methodological discrepancy may restrict the translational medicine value of the existing evidence, as it does not fully reflect the therapeutic challenges encountered in clinical practice. Future research should therefore prioritize a more comprehensive exploration of these downstream effects, such as DNA binding and epigenetic regulation, while focusing on administration strategies that are more clinically relevant to thoroughly assess the therapeutic potential of monomeric compounds in AP therapy.

Despite their potent regulatory effects on STAT signaling, the clinical translation of these monomers is largely dictated by their pharmacokinetic (PK) profiles. A primary challenge shared by polyphenols (e.g., curcumin, baicalein), terpenoids (e.g., limonin, picroside II) and anthraquinones (rhein) is limited oral bioavailability, often resulting from poor aqueous solubility, extensive first-pass metabolism, or active efflux mediated by P-glycoprotein (P-gp) 111-114. Despite these barriers, monomers achieve therapeutic thresholds in the pancreas through several distinct mechanisms. Many monomers exist primarily as metabolites *in vivo*, which may serve as the actual pharmacodynamic basis. Rhein is rapidly absorbed and converted into glucuronide and sulfate forms, which undergo enterohepatic circulation; its linear PK properties ensure consistent systemic exposure despite low solubility 113,115. Similarly, rutaecarpine undergoes extensive phase I and II metabolism (hydroxylation and conjugation), maintaining its potent induction of hepatic CYP1A2 even when parent plasma levels are nearly undetectable (below 10 ng/mL) 116,117. In contrast, kinsenoside is characterized by rapid assimilation and clearance, with its stability significantly influenced by chemical hydrolysis of its lactone group rather than enzymatic degradation 118,119. Effective modulation of the JAK/STAT axis requires drugs to penetrate the blood-pancreatic barrier. While colchicine (derived from colchicum autumnale) exhibits a low oral bioavailability (< 8%), its high volume of distribution suggests extensive tissue uptake, though its use is constrained by a narrow therapeutic window and potential hepatotoxicity 120. For rhein, structural modifications have proven successful in enhancing targeting; specifically, ester and amide derivatives like HPDM-rhein have demonstrated a superior ability to cross the blood-pancreatic barrier and accumulate in the lungs and pancreas, directly addressing the systemic complications of AP 113. To overcome the inherent PK limitations of natural products, innovative delivery systems and synergistic pharmacological strategies are employed. Nano-formulations, such as PEGylated liposomes for curcumin and PLGA-nanoparticles for lutein, can increase the AUC by 3- to 77-fold 121,122. Furthermore, glycyrrhizin acts as a complex PK modulator; it can reduce the systemic exposure of co-administered drugs like paeoniflorin via P-gp induction, yet it may also delay the clearance of others, leading to increased toxicity 123,124. Conversely, kinsenoside shows minimal interference with CYP450 enzymes, suggesting a higher safety profile for combination therapies 118. In summary, the translational potential of these monomers relies on a multidimensional PK strategy: utilizing metabolic activation, leveraging vascular permeability, and employing advanced delivery platforms to ensure that STAT-modulating agents reach sufficient concentrations within the diseased pancreatic microenvironment.

### 3.3 Chemical drugs

Glucocorticoids, including dexamethasone (DEX), are widely employed for their potent anti-inflammatory and immunosuppressive effects. In both MAP and SAP rat models, DEX has been demonstrated to attenuate pancreatic injury by downregulating inflammatory cytokines and chemokines, disrupting STAT3-DNA binding, and suppressing the MAPK/NF-κB/STAT3 signaling pathway 125. Compared to the model group, DEX administration resulted in pronounced reductions in MAPK kinase activity and IL-1β levels during the early phase of AP (3 hours), whereas NF-κB activity and STAT3 expression were significantly suppressed at later stages in MAP (12 hours) and at the early phase in SAP (3 hours), respectively, indicating a time-dependent therapeutic effect 125. Notably, another study demonstrated that DEX mitigates SAP-associated lung injury by downregulating intercellular adhesion molecule-1, JAK2, STAT3, and pro-inflammatory cytokines, thereby suppressing the JAK2/STAT3 signaling axis 126. These findings collectively suggest that DEX may protect against both pancreatic and extra-pancreatic organ damage in a temporally regulated manner during AP progression.

Thiamine (vitamin B1, VB1) is an essential micronutrient that plays a pivotal role in cellular energy metabolism. PACs primarily depend on thiamine transporter-1 and -2 (THTR-1 and THTR-2) for VB1 uptake from the circulation. Exposure of PACs to elevated levels of inflammatory cytokines (IL-6, IL-1β, TNF-α) for 24 hours has been shown to markedly impair VB1 uptake 127. Treatment with the STAT3-specific inhibitor S3I-201 downregulated p-STAT3 (Tyr705) levels, thereby inhibiting the IL-6/STAT3/THTR pathway and restoring IL-6-induced reductions in VB1 uptake in 266-6 cells 127. These findings indicate that modulation of the STAT3 pathway may represent a potential strategy to enhance energy metabolism in PACs, although *in vivo* validation remains lacking.

Alprostadil, a prostaglandin E1 analog, is widely used in the management of urological disorders and has recently attracted attention for its potential therapeutic application in inflammatory conditions. In an ARG-induced SAP rat model, levels of malondialdehyde (MDA), myeloperoxidase (MPO), and pro-inflammatory cytokines (IL-6, IL-1β, TNF-α) were significantly elevated, whereas superoxide dismutase activity was reduced, reflecting exacerbated oxidative stress and inflammatory responses 128. Administration of alprostadil markedly reversed these changes and concurrently downregulated the expression of JAK2 and STAT3 proteins. Notably, the therapeutic efficacy of alprostadil was comparable to that of the JAK2-specific inhibitor AG490, indicating that alprostadil mitigates SAP progression via inhibition of the JAK2/STAT3 signaling pathway 128.

Carvedilol, a non-selective β-adrenergic blocker, has been shown to exert protective effects by attenuating oxidative stress and inflammatory responses. In an ARG-induced AP rat model, oxidative stress markers, including MDA, were significantly elevated, whereas antioxidant factors such as glutathione and catalase were decreased. Concurrently, inflammatory mediators, including C-reactive protein, IL-1β, TNF-α, and MPO, were markedly increased 129. Administration of carvedilol ameliorated these alterations and markedly downregulated the mRNA expression of PAP2 and platelet-activating factor, as well as the protein levels of NF-κB p65, MAPK-p38, and STAT1. These findings indicate that carvedilol mitigates SAP progression primarily through inhibition of the MAPK/NF-κB/STAT1 signaling pathway 129.

According to the 2025 revised guidelines, the routine management of AP mainly comprises analgesia, fluid resuscitation, and nutritional support, as no specific pharmacological therapy has been firmly established 130. Nonetheless, recent preclinical studies have demonstrated that several western drugs, including DEX, VB1, alprostadil, and carvedilol, can attenuate inflammation, enhance microcirculation, and modulate metabolic dysfunction in AP models, primarily through modulation of STATs-related signaling pathways. These findings offer valuable experimental evidence for optimizing therapeutic windows and designing intervention strategies. Nevertheless, current research remains largely confined to STAT3-associated pathways, and the functional roles of other STATs family members remain insufficiently characterized. Notably, except for DEX pharmacological studies, which employed both MAP and SAP models at multiple time points, most investigations are limited by the use of single models and static observational time points. Such an approach does not allow a systematic evaluation of drug effects across diverse etiologies and disease stages. Importantly, the lack of established positive control drugs not only diminishes the comparability and persuasiveness of the results but also underscores the current absence of targeted therapeutics for SAP. Furthermore, since these drugs have not been incorporated into clinical practice for AP, their efficacy and safety profiles require further validation through large-scale prospective studies and clinical trials. Consequently, their inclusion in international guidelines and standard treatment protocols remains a long-term objective.

## 4. Clinical Evidence

**Figure 4:**
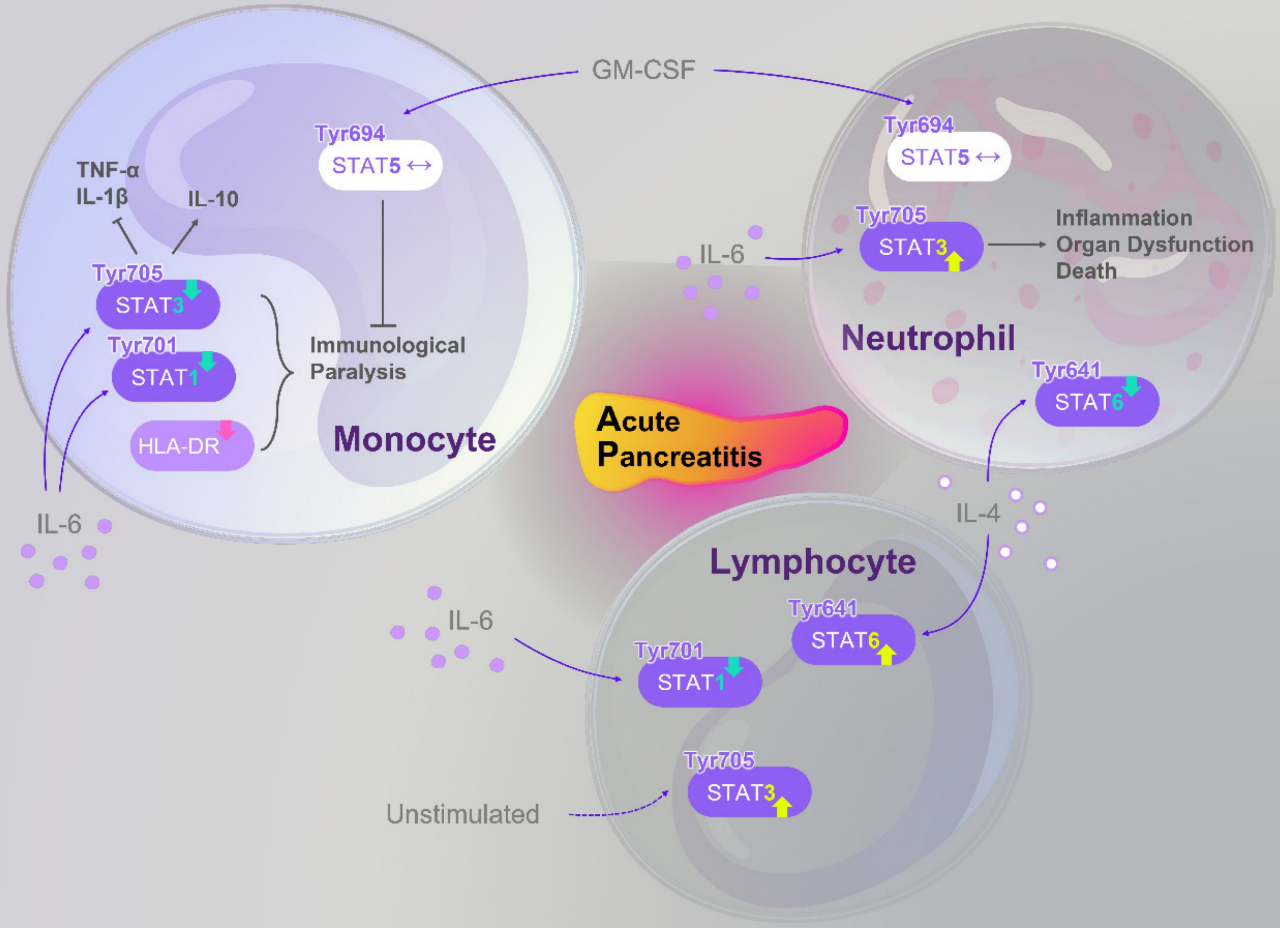
** Abnormal alterations of the STATs family in immune cells of AP patients.** Members of the STATs family exhibit dysregulated signaling in monocytes, lymphocytes, and neutrophils of AP patients, further modulating immune function and inflammation. In monocytes, IL-6 stimulation results in reduced levels of p-STAT3 (Tyr705) and p-STAT1 (Tyr701). This reduction, combined with downregulation of HLA-DR expression, contributes to an “immunoparalysis” phenotype. However, normal p-STAT5 (Tyr694) expression under GM-CSF stimulation can partially alleviate this immunosuppressed state. Moreover, decreased p-STAT3 (Tyr705) disrupts the balance between pro-inflammatory cytokines (TNF-α, IL-1β) and the anti-inflammatory cytokine IL-10. In neutrophils, p-STAT5 (Tyr694) remains largely unchanged upon GM-CSF stimulation; in contrast, IL-6 induces an increase in p-STAT3 (Tyr705), whereas IL-4 leads to decreased p-STAT6 (Tyr641). These alterations are associated with inflammation, organ dysfunction, and mortality in AP patients. In lymphocytes, p-STAT3 (Tyr705) remains constitutively active under unstimulated conditions; following IL-6 stimulation, p-STAT1 (Tyr701) activation is impaired, while IL-4 stimulation enhances p-STAT6 (Tyr641) activation. This signaling imbalance contributes to an increased risk of secondary infections in AP patients. Abbreviations: AP, acute pancreatitis; GM-CSF, granulocyte-macrophage colony-stimulating factor; HLA-DR, human leukocyte antigen-DR; IL, interleukin; STAT, signal transducer and activator of transcription; TNF-α, tumor necrosis factor-α; Tyr, tyrosine.

Beyond experimental and pharmacological studies, clinical evidence highlights the pivotal role of STATs signaling in AP pathophysiology. Altered STATs activity has been observed in immune cells of SAP patients, correlating with inflammatory status, disease progression, and systemic complications. A concise overview of these findings is presented in **Figure 4**, which serve as references for the subsequent discussion.

### 4.1 Abnormal STATs signaling in immune cells of SAP patients

Current clinical research on STAT signaling in AP is predominantly based on observational profiling, with direct mechanistic insights remaining exceptionally rare. Based on observational clinical data, studies have demonstrated that the STATs family exhibits a certain common regulatory pattern in different immune cells (lymphocytes, monocytes, and neutrophils) of patients with SAP complicated by MODS. As upstream signaling molecules of the STATs family, p-ERK1/2 (Thr202/Tyr204) and NF-κB p-p65 (Ser529) are downregulated in lymphocytes, monocytes, and neutrophils isolated from SAP patients 131-133. Compared with healthy individuals, the expression level of p-STAT1 (Tyr701) in lymphocytes and monocytes of SAP patients is significantly reduced following stimulation with IL-6 131,132. However, no significant changes are observed in the level of MAPK-p-p38 (Thr180/Tyr182) in monocytes and neutrophils 132,133. Similarly, no significant alteration is detected in the level of p-STAT5 (Tyr694) in these cells following stimulation with granulocyte-macrophage colony-stimulating factor 132,133. These shared alterations suggest that the STATs family and its associated signaling pathways may exhibit global regulatory abnormalities in the immune dysregulation of SAP.

In terms of cell type-specific manifestations, lymphocytes exhibit distinct abnormalities in STATs signaling. Under basal conditions without exogenous cytokine stimulation, the proportion of p-STAT3 (Tyr705)-positive lymphocytes in SAP patients reaches 42.0%±4.7%, which is significantly higher than that in healthy controls (2.6%±0.1%, P < 0.001), and STAT3 shows sustained activation 131—a phenomenon not observed in monocytes 132. In addition, lymphocytes display an imbalance between STAT1 and STAT6 signaling, characterized by downregulation of p-STAT1 (Tyr701) upon IL-6 stimulation and upregulation of p-STAT6 (Tyr641) upon IL-4 stimulation 131. Given that STAT1 is a key regulator of Th1 cell differentiation and effector function, whereas STAT6 is critical for Th2 cell differentiation, this signaling imbalance reflects suppression of Th1-mediated cellular immunity and activation of Th2-mediated humoral responses, thereby creating a gap in cellular immune defense and increasing the risk of secondary infections in SAP patients.

In contrast to the activation pattern of STAT3 in lymphocytes, monocytes exhibit distinct STATs signaling features upon IL-6 stimulation. Compared with healthy controls, the proportion of p-STAT3 (Tyr705)-positive monocytes in SAP patients is significantly reduced (75.3%±5.7% vs 97.3%±0.3%) 132. STAT3 activation in monocytes normally promotes the production of the anti-inflammatory cytokine IL-10 and suppresses pro-inflammatory cytokines such as TNF-α and IL-1β 134,135. Disruption of this activation impairs the anti-inflammatory function of monocytes, rendering the STAT3 signaling pathway in these cells more severely compromised than in lymphocytes. Notably, monocytes from SAP patients also show reduced p-STAT1 (Tyr701) levels and marked downregulation of human leukocyte antigen-DR expression (55.0%±4.1% vs 93.1%±3.4%, P < 0.001) compared with healthy controls 132. Together with weakened STAT3 phosphorylation, these alterations constitute a hallmark of monocyte “immunoparalysis”, reflecting diminished responsiveness to pathogens and damage signals. This immunological state, typically observed in patients classified as SAP according to the Atlanta classification 132, represents a key factor contributing to the development of intra-abdominal abscesses and sepsis, which occurred in 69.2% of the patients within the observed sample 132.

Further comparison of STATs alterations across different immune cell types reveals opposing trends in monocytes and neutrophils regarding p-STAT3 (Tyr705) levels. In SAP patients, the proportion of p-STAT3-positive neutrophils following IL-6 stimulation was significantly increased compared with healthy controls (38.1%±8.4% vs 10.7%±2.2%, P = 0.016) 133, suggesting a potential role in inflammation and mortality-associated signaling. Additionally, neutrophil STAT6 abnormalities differed markedly from those in lymphocytes. Upon IL-4 stimulation, p-STAT6 (Tyr641) fluorescence intensity in neutrophils from SAP patients was significantly reduced relative to healthy controls 133. Given that STAT6 indirectly regulates anti-inflammatory responses and the clearance of apoptotic cells, its impaired activation may compromise inflammation resolution in SAP patients. Clinical data show reduced STAT6 in SAP, but it remains unclear whether therapeutically restoring STAT6 might paradoxically interfere with phagocytic clearance of translocated bacteria while suppressing inflammation.

### 4.2 Roles of STATs signaling in severity evaluation, prognosis prediction, and complication risk of AP

To ensure clinical diagnostic rigor, the studies discussed herein primarily defined the severity of AP according to the Atlanta or revised Atlanta classification systems 132,136,137. Acting as observational biomarkers, Turunen *et al.* demonstrated that the dynamic evolution of STAT3 and STAT1 phosphorylation reflects differences in clinical outcomes among AP patients. At the STAT3 level, upon admission, monocytes and neutrophils from AP patients exhibited elevated constitutive STAT3 phosphorylation compared with healthy controls, regardless of the presence of persistent organ dysfunction (OD) 136. In this context, persistent OD was rigorously defined by the Revised Atlanta Classification as a Modified Marshall Score (MMS) ≥ 2 lasting more than 48 hours 136. Moreover, IL-6-stimulated p-STAT3 (Tyr705) levels in monocytes from both patient groups (OD- group representing moderately severe AP; OD+ group representing severe AP) were lower than those in healthy controls 136. Between days 5 and 8 after admission, constitutive STAT3 phosphorylation in both monocytes and neutrophils declined in both groups, although monocyte levels remained higher than in controls (P = 0.007) 136. Notably, IL-6-stimulated monocyte p-STAT3 (Tyr705) in the OD- group showed partial recovery (still below control levels), suggesting a gradual restoration of IL-6 responsiveness, which correlates with the absence of persistent OD (MMS < 2) and zero 30-day mortality in this group 136. At the STAT1 level, IL-6-stimulated monocyte p-STAT1 (Tyr701) was lower in both patient groups compared with controls upon admission, while lymphocytes showed no significant difference 136. This reduced monocyte p-STAT1 (Tyr701) expression persisted at days 5-8, potentially due to miR-30a-mediated targeting of STAT1 138 or downregulation of the IL-6 receptor 139, reflecting sustained impairment of monocyte anti-infective signaling pathways in AP patients.

From the perspective of observational biomarkers for disease severity and complication prediction, the clinical relevance of aberrant STAT3, STAT1, and STAT6 phosphorylation is increasingly evident. Regarding STAT3, basal p-STAT3 (Tyr705) levels in AP patients are significantly higher than in healthy controls and correlate positively with disease severity as assessed by standardized scoring systems 140,141. In patients sampled before the onset of OD, elevated p-STAT3 (Tyr705) levels can predict the development of persistent OD, defined by a Modified Marshall Score ≥ 2 or an acute increase in the Sequential Organ Failure Assessment score of two or more points 140,141. In the study by Ezzat *et al.* 137, patients with necrotizing AP exhibited significantly higher levels of STAT3 mRNA expression compared with those with non-necrotizing AP. This elevated STAT3 expression was found to correlate with several indicators of disease severity, including the SIRS and the computed tomography severity index, as well as the presence of pancreatic necrosis 137. However, it is important to note that STAT3 mRNA levels did not demonstrate a significant correlation with BISAP scores in this cohort. Furthermore, while receiver operating characteristic curve analysis was employed to assess its diagnostic potential, STAT3 mRNA did not show statistical significance as an independent predictor of pancreatic necrosis, yielding a *P* value of 0.141 137. Instead, the analysis focused on its ability to identify the combination of moderate and severe AP, although the resulting area under the curve of 0.64 also lacked statistical significance. These findings suggest that while STAT3 mRNA levels reflect certain inflammatory and necrotic processes in AP, their current utility as standalone prognostic biomarkers remains limited and requires further clinical validation.

Moving beyond observational correlations to mechanistic causality and therapeutic interventions, genetic and pharmacological studies provide direct evidence of STATs signaling in driving clinical outcomes. For instance, the risk allele C of the γ-glutamyltransferase 1 single nucleotide polymorphism rs5751901 increases the risk of post-endoscopic retrograde cholangiopancreatography pancreatitis by enhancing STAT3 activation 142, whereas QYG combined with DEX can effectively reduce complication incidence in AP patients by inhibiting the IL-6/STAT3 pathway, highlighting p-STAT3 as a potential preventive and therapeutic target 84. Returning to prognostic biomarkers, changes in STAT1 display an opposite trend to STAT3. IL-6-stimulated p-STAT1 (Tyr701) levels in monocytes and lymphocytes decrease with increasing AP severity 140,141, which may result from downregulation of the IL-6 receptor 139 or reduced phosphorylation of glycoprotein 130 143. This hypo-responsiveness of STAT1 can predict the risk of secondary infections, providing a reference for early identification of high-risk patients. In addition, IL-4-stimulated p-STAT6 (Tyr641) levels in monocytes and neutrophils from AP patients are lower than in healthy controls 141. Although p-STAT6 is not significantly associated with disease severity, in patients with existing OD, low p-STAT6 (Tyr641) can predict persistent OD, offering a novel molecular marker for assessing prognosis of organ failure 141. In summary, the integration of molecular STAT signaling profiles with established clinical metrics like BISAP, APACHE II, and Marshall scores offers a more comprehensive framework for early risk stratification and prognosis prediction in AP.

Current clinical studies indicate that STATs signaling plays a crucial role in immune regulation during AP. However, several limitations remain. First, while specific alterations have been observed in lymphocytes, monocytes, and neutrophils, the complex correlations and signaling crosstalk between these distinct immune cell populations remain to be elucidated. Research remains focused on STAT1, STAT3, and STAT6, while the roles and mechanisms of STAT2, STAT4, and STAT5 remain largely unexplored. Second, current clinical evidence is disproportionately skewed toward peripheral immune cell profiling, whereas direct investigations into the STAT signaling dynamics within human pancreatic parenchymal cells remain nearly non-existent. This lack of “*in-situ*” clinical data hinders our understanding of the direct interplay between immune dysregulation and acinar/ductal injury. Third, the vast majority of existing data is derived from observational profiling, with a critical shortage of interventional studies, such as pharmacological modulation or genetic risk-allele analysis (e.g., SNP studies), that could definitively establish mechanistic causality. Future investigations should therefore aim to: (1) expand research beyond STAT1/3 to include the entire STAT family, focusing specifically on the coordinated crosstalk between immune and parenchymal cells to establish a holistic map of the AP microenvironment, (2) promote the development and implementation of rapid bedside detection technologies, such as integrated microfluidic chips, and (3) design large-scale, multicenter, stratified, and longitudinal clinical studies, coupled with rigorous bidirectional validation between basic and clinical research, to facilitate the transition from descriptive inflammatory profiling (observational) to precise immune modulation (mechanistic).

## 5. Translational Challenges and Precision Therapeutic Strategies

The functional duality of STATs, particularly STAT3, represents a significant hurdle in translating experimental findings into clinical efficacy. The recent clinical failure of the STAT3 antisense oligonucleotide danvatirsen underscores the risks of global inhibition, which can lead to unintended immune suppression 144. Because STAT3 is indispensable for maintaining intestinal epithelial homeostasis and supporting pancreatic acinar cell regeneration during the resolution phase, indiscriminate blockade may inadvertently compromise innate repair mechanisms or exacerbate the risk of secondary infections 145,146. Furthermore, the optimization of the therapeutic window through strategic timing is equally critical. Because the IL-22/STAT3 axis promotes essential anti-apoptotic and pro-regenerative effects during the resolution phase, a “one-size-fits-all” inhibitory approach may inadvertently delay tissue repair 147,148. Consequently, future therapeutic efforts must move toward cell-type, organelle, or pathway selective modulation through strategic timing. A biphasic model that inhibits pro-inflammatory STATs signaling during the hyperacute phase while selectively activating restorative pathways, such as through IL-22 mRNA loaded lipid-nanoparticles, may optimize the therapeutic window 149. Advanced delivery systems further offer a promising solution to these compartmentalized roles; for instance, fibroblast activation protein alpha targeted nanocarriers or pH responsive micelles can concentrate therapeutic agents within activated fibroblasts or damaged acinar cells while sparing systemic immune functions 150,151. Such technologies are especially vital for TCM monomers, as their clinical utility is often hampered by low oral bioavailability and rapid systemic metabolism 152.

## 6. Concluding Remarks and Future Directions

In summary, this review has synthesized the critical role of the STATs family as a regulatory hub that orchestrates the complex transition from local injury to systemic inflammation in AP. While these insights provide a robust conceptual framework, translating them into therapeutic reality requires overcoming the current reliance on idealized rodent models 153,154 and the limitations of correlational protein expression analysis. To bridge this gap and establish definitive causal relationships, the field must now transition from broad observation toward genetic rescue experiments and cell-type specific investigations.

Moving forward, the field must test specific mechanistically driven hypotheses to bridge the gaps identified in the preceding sections. Primarily, it remains to be determined whether STAT3 activation in pancreatic stellate cells during early AP contributes to ductal pressure regulation by altering pancreatic architectural stiffness 155. Simultaneously, research should investigate if pharmacological STAT6 activation, while intended to promote inflammation resolution, might inadvertently compromise the host ability to clear translocated gut bacteria 156,157. Thirdly, studies should explore whether the STAT4 mediated Th1/Th17 balance dictates the transition from acute injury to chronic fibro inflammatory progression 158. Finally, given that STAT1 and STAT3 activation drives premature protease activation in acinar cells 39, it remains to be seen whether activating distinct isoforms, such as STAT5 in ductal cells, can mitigate bicarbonate secretion impairment and thereby restore the alkaline environment necessary to prevent this injury.

While TCM monomers and synthetic modulators show significant potential, more rigorous preclinical studies and higher levels of clinical evidence are necessary to firmly establish their efficacy 159. Mechanistic investigations utilizing spatiotemporal analysis will be essential to clarify stage-specific roles, while pharmacological research must focus on dose-response relationships and extra-pancreatic organ protection. Ultimately, integrating these molecular insights with precision delivery technologies that can sense the pathological microenvironment will be essential to realize individualized therapy and improve patient outcomes in AP.

## Figures and Tables

**Table 1:** Agonist modulating the STATs pathway for AP therapy.

Compounds	Mechanism	Concentration	Cell/Animal Study	Function	Ref
CpG 1826	↑TLR9, ↑IL-6, ↑p-STAT3	4 mg/kg i.p.	CER-AP mice	Regulation of the TLR9/IL-6/STAT3 pathway to modulate immune responses, and alleviate pancreatic and intestinal tissue injury	59
Garcinone D	↑p-JAK2, ↑p-STAT3 (Tyr705)	10 μM	CER-AP AR42J cells	Activating the JAK2/STAT3 pathway to exacerbate pancreatitis	65
Recombinant IFN-γ	↑Ac-STAT1	10⁴ U per mouse, s.c.	CER-AP mice	Targeting the Ac-STAT1 signaling to alleviate pancreatic injury and modulate immune responses	21
Recombinant IL-10	↑p-STAT3 (Tyr705)	10 μg/kg i.v.	NaT-SAP rats	Modulation of the IL-10/STAT3 pathway to alleviate pancreatic injury	80
Recombinant IL-22	↑p-STAT3 (Tyr705)	200 ng per mouse, i.p. or s.c. *in vivo* 20 ng/mL *in vitro*	CDE-AP mice NaT-SAP mice ARG-SAP mice CER-AP PACs	Modulating the IL-22/STAT3/Reg3 pathway to regulate immune responses and attenuate pancreatic, pulmonary, and intestinal injury	37 38 45 78
Recombinant MFG-E8	↑p-STAT3 (Ser727), ↑p-STAT3 (Tyr705)	5, 10, 20 μg/kg i.p. *in vivo* 20, 100 ng/mL *in vitro*	ARG-SAP mice, CER-AP mice, CER+LPS-AP mice CER+LPS-AP AR42J cells	Targeting the FAK/STAT3 pathway to alleviate pancreatic injury	43

Abbreviations: Ac, Acetylated; AP, acute pancreatitis; ARG, arginine; CDE, DL-ethionine; CER, caerulein; FAK, focal adhesion kinase; IL, interleukin; i.p., intraperitoneal; i.v., intravenous; LPS, lipopolysaccharide; MFG-E8, milk fat globule epidermal growth factor 8; NaT, sodium taurocholate; PACs, pancreatic acinar cells; p-STAT, phosphorylated signal transduction and transcriptional activation factor; Reg3,Regenerating islet-derived protein 3; SAP, severe acute pancreatitis; s.c., subcutaneously; Ser, serine; STAT, signal transduction and transcriptional activation factor; TLR9, toll-like receptor 9; Tyr, tyrosine.

**Table 2:** Antagonist targeting the STATs pathway for AP treatment.

Compounds	Mechanism	Concentration	Cell/Animal Study	Function	Ref
AG490	↓p-JAK2 (Tyr1007/1008), ↓p-STAT3 (Ser727), ↓p-STAT3 (Tyr705)	8 mg/kg i.p. or i.v. *in vivo* 0.02 g/kg i.p. *in vivo* 50 μM *in vitro*	NaT-SAP rats CER-AP mice CER-AP AR42J cells	Inhibiting the JAK2/STAT3 and IL-6/STAT3 pathways to modulate immune responses and attenuate pancreatic and hepatic injury	23 46 20 34 25
A17pro	↓p-STAT3 (Tyr705)	1 mg/kg i.p.	CER-AP mice NNK-AP mice	Suppression of the IL-6/STAT3 pathway to alleviate pancreatic injury and modulate immune responses	53
Anti-viral cocktail	↓TLR9, ↓IL-6, ↓p-STAT3	30 mg/kg ribavirin, 10 mg/kg lamivudine, 20 mg/kg acyclovir i.g.	CER-AP mice	Targeting the TLR9/IL-6/STAT3 pathway to modulate immune responses, and alleviate pancreatic and intestinal tissue injury	59
APTSTAT3-9R	↑p-FAK, ↓p-STAT3 (Ser727)	30 μM	CER+LPS-AP AR42J cells	Modulation of the FAK/STAT3 pathway to alleviate pancreatic injury	43
Cilengitide	↓p-FAK, ↓p-STAT3 (Ser727), ↓p-STAT3 (Tyr705)	20 mg/kg i.p.	ARG-SAP mice	Targeting the FAK/STAT3 pathway to alleviate pancreatic injury	43
DADS	↑PPAR-γ, ↑SOCS3, ↓p-STAT3(Tyr705)	200 μg/kg i.p.	CER-AP mice	Inhibition of the PPAR-γ/SOCS3/STAT3 pathway to attenuate pancreatic and pulmonary tissue injury	55
Dexamethasone	↓STAT3	10^-^⁷ M	PACs from NaT-SAP rats	Suppression of the STAT3 signaling to alleviate pancreatic injury	60
Diphenyleneiodonium	↓p-JAK2 (Tyr1007/1008), ↓p-STAT3 (Tyr705)	10 μM	CER-AP AR42J cells	Targeting the JAK2/STAT3 pathway to alleviate pancreatic injury	25
EX527	↓SIRT1, ↑Ac-STAT3	20 mg/kg i.p.	CER-AP mice	Modulation of the IL-6/STAT3 pathway and SIRT1/STAT3 pathway to alleviate pancreatic injury and modulate immune responses	56
Fedratinib	↓p-JAK2, ↓p-STAT3 (Tyr705)	3 nM	CER-AP AR42J cells	Inhibiting the JAK2/STAT3 pathway to mitigate pancreatitis	65
HJC0152	↓STAT1, ↓p-STAT1 (Tyr701), ↓p-STAT3 (Tyr705)	10 μM	CVB3-AP 266-6 cells	Suppression of STAT3 signaling to enhance STAT1 activation, mitigating pancreatic and cardiac damage	44
N-acetylcysteine	↓STAT3	50 mg/kg i.p.	BPDO-AP rats NaT-SAP rats	Targeting the STAT3 signaling to alleviate pancreatic injury	57
ODN2088	↓TLR9, ↓p-STAT3	4 mg/kg i.p.	CER-AP mice	Inhibition of the TLR9/IL-6/STAT3 pathway to modulate immune responses, and alleviate pancreatic and intestinal tissue injury	59
PF-00562271	↓p-FAK, ↓p-STAT3 (Ser727)	1 μM	CER+LPS-AP AR42J cells	Suppression of the FAK/STAT3 pathway to alleviate pancreatic injury	43
PP2	↓p-Src, ↓p-ERK, ↓p-JNK, ↓p-STAT3 (Tyr705)	0.5, 1, 1.5 mg/kg i.p. *in vivo* 1, 10 μM *in vitro*	substance P-AP PACs CER- AP mice	Targeting the SFKs/STAT3 pathway to alleviate pancreatic injury	29
Peptide YY	↓STAT1, ↓STAT3	500 pM	TNF-α-AP AR42J cells	Modulation of the STAT1 and STAT3 signaling to alleviate pancreatic injury	39
(R)-TML104	↑SIRT1, ↓Ac-STAT3, ↓p-STAT3 (Tyr705), ↓IL-6	5, 10, 20 mg/kg i.p.	CER-AP mice	Targeting the IL-6/STAT3 pathway and SIRT1/STAT3 pathway to alleviate pancreatic injury and modulate immune responses	56
Ruxolitinib	↓p-JAK2, ↓p-STAT3	180 mg/kg i.g.	NaT-SAP rats	Targeting the JAK2/STAT3 pathway to alleviate pancreatic and intestinal tissue injury	82
S3I-201	↓p-STAT3 (Tyr705)	5 mg/kg i.p.	NaT-SAP rats	Targeting the IL-10/STAT3 pathway to alleviate pancreatic injury	80
SEW2871	1. ↓p-STAT3	20 mg/kg i.g.	CER-AP mice	Inhibition of the STAT3 signaling to alleviate pancreatic injury and modulate immune responses	61
sgp130Fc	↓p-STAT3 (Tyr705), ↓p-STAT3 (Ser727)	0.3 mg/mouse i.p.	CER-AP mice	Suppression of the IL-6/STAT3 pathway to attenuate pancreatic and pulmonary tissue injury	31
Stattic	↓p-STAT1 (Tyr701), ↓p-STAT3 (Tyr705), ↓p-JAK2	3.75 mg/kg i.p. *in vivo* 2.5, 5 μM *in vitro*	NaT-SAP rats CVB3-AP 266-6 cells CER-AP AR42J cells	Inhibiting the JAK2/STAT3 pathway to alleviate pancreatic, intestinal and myocardial injury	44 82 65

Abbreviations: Ac, acetylated; AP, acute pancreatitis; A17pro, ADAM17 Prodomain Inhibitor; ARG, arginine; BPDO, bile-pancreatic duct obstruction; CER, caerulein; CP96345, (2S,3S)-cis-2-(diphenylmethyl)-N-((2-methoxyphenyl)-methyl)-1-azabicyclo(2.2.2.)-octan-3-amine; FAK, focal adhesion kinase; IL, interleukin; i.g., intragastric; i.p., intraperitoneal; i.v., intravenous; JAK, janus kinase; LPS, lipopolysaccharide; MFG-E8, milk fat globule epidermal growth factor 8; NaT, sodium taurocholate; NNK, nicotine-derived nitrosamine ketone; PACs, pancreatic acinar cells; PP2, 4-amino-5-(4-chlorophenyl)-7-(t-butyl) pyrazolo [3,4-D] pyrimidine; PPAR-γ, peroxisome proliferator-activated receptor gamma; p-STAT, phosphorylated signal transduction and transcriptional activation factor; (R)-TML104, (R)-4,6-dimethoxy-3-(4-methoxy phenyl)-2,3-dihydro-1H-indanone; SAP, severe acute pancreatitis; Ser, serine; SFK, Src family kinase; SIRT1, sirtuin 1; SOCS3, suppressor of cytokine signaling 3; STAT, signal transduction and transcriptional activation factor; TLR9, toll-like receptor 9; TNF-α, tumor necrosis factor alpha; Tyr, tyrosine.

**Table 3:** Herbal formulas modulating the STATs pathway for AP therapy.

Formulas	Herbs	Preparation Method	Standardization	Cell/Animal Study	Pathways	Mechanism	Ref
Da Cheng Qi Decoction	*Rheum palmatum* L. (Da Huang), *Magnolia officinalis* Rehd. et Wils. (Hou Po), *Citrus aurantium* L. (Zhi Shi), and *Natrii Sulfas* (Mang Xiao)	Water decoction with ethanol precipitation Reconstituted spray-dried powders	HPLC fingerprinting Chinese Pharmacopoeia standards	CER-AP AR42J cells NaT-AP rats	JAK2/STAT3 signaling pathway	↓p-JAK2, ↓p-STAT3 (Tyr705)	65 82
Chaiqin Chengqi Decoction	*Bupleurum chinense* DC. (Chai Hu), *Scutellaria baicalensis* Georgi (Huang Qin), *Magnolia officinalis* Rehd. et Wils. (Hou Po), *Citrus aurantium* L. (Zhi Shi), *Gardenia jasminoides* Ellis (Zhi Zi), *Rheum palmatum* L. (Da Huang), *Natrii Sulfas* (Mang Xiao), *Corydalis yanhusuo* W.T. Wang (Yan Hu Suo), *Ligusticum chuanxiong* Hort. (Chuan Xiong), and *Aucklandia lappa* Decne. (Mu Xiang)	Water decoction	Hospital-authenticated raw materials	CER-AP AR42J cells CER-AP rats	JAK2/STAT3 signaling pathway	↓p-JAK2, ↓p-STAT3	83
Qingyi Granules	*Scutellaria baicalensis* Georgi (Huang Qin)*, Gardenia jasminoides* Ellis (Zhi Zi)*, Corydalis yanhusuo* W.T. Wang (Yan Hu Suo)*, Bupleurum chinense* DC. (Chai Hu)*, Rheum palmatum* L. (Da Huang)*, Paeonia lactiflora* Pall. (Bai Shao)*, Natrii Sulfas* (Mang Xiao), and *Aucklandia lappa* Decne. (Mu Xiang)	Standardized granules; reconstituted for intragastric administration	UHPLC-QE-MS serum pharmacochemistry	NaT-AP rats	IL-6/STAT3 signaling pathway	↓IL-6, ↓p-STAT3	84
Chaihuang Qingyi Huoxue Granule	*Bupleurum chinense* DC. (Chai Hu)*, Magnolia officinalis* Rehd. et Wils. (Hou Po), *Paeonia lactiflora* Pall. (Chi Shao), *Rheum palmatum* L. (Da Huang), *Prunus persica* (L.) Batsch (Tao Ren), *Salvia miltiorrhiza* Bge. (Dan Shen), *Glycyrrhiza uralensis* Fisch. (Gan Cao), *Corydalis yanhusuo* W.T. Wang (Yan Hu Suo)*, Astragalus membranaceus* (Fisch.) Bge. (Huang Qi),* Scutellaria baicalensis* Georgi (Huang Qin)*, Citrus aurantium* L. (Zhi Shi), *Gardenia jasminoides* Ellis (Zhi Zi),* Paeonia lactiflora* Pall. (Bai Shao)*, Taraxacum mongolicum* Hand.-Mazz.* (Pu Gong Ying)*	Commercial granules; saline decoction	Standardized commercial formulation	NaT-AP rats	PI3K/AKT signaling pathway	↓p-PI3K, ↓p-AKT	85
Qingyi Decoction	*Rheum palmatum* L. (Da Huang), *Bupleurum chinense* DC. (Chai Hu)*, Aucklandia lappa* Decne. (Mu Xiang), *Corydalis yanhusuo* W.T. Wang (Yan Hu Suo)*, Paeonia lactiflora* Pall. (Bai Shao)*, Scutellaria baicalensis* Georgi (Huang Qin)*, Coptis chinensis* Franch. (Huang Lian)	Water decoction; lyophilized powder	UHPLC-Orbitrap-MS/MS profiling	CER-AP mice	NF-κB/NLRP3 signaling pathway	↓NF-κB, ↓NLRP3	86
Da-Huang-Fu-Zi-Tang	*Rheum palmatum* L. (Da Huang), *Aconitum carmichaelii* Debx. (Fu Zi), *Asarum heterotropoides* F. Schmidt (Xi Xin)	Water decoction; vacuum concentration	HPLC-ESI-MS/UV profiling	NaT-AP rats	JAK2/STAT3 signaling pathway	↓p-JAK2, ↓p-STAT3	87
Shenmai Injection	*Panax ginseng* C.A. Mey. (Ren Shen) and *Ophiopogon japonicus* (L.f.) Ker Gawl. (Mai Dong)	Commercial patent drug	UHPLC-QTOF/MS analysis	NaT-AP 266-6 cells NaT-AP mice	IL-6/STAT3 signaling pathway	↓IL-6, ↓p-STAT3 (Tyr705)	89
*Rheum palmatum* L. and *Salvia miltiorrhiza* Bge.	*Rheum palmatum* L. (Da Huang) and *Salvia miltiorrhiza* Bge. (Dan Shen)	DH-DS herbal pair (1:1 ratio); oral gavage (crude drug equivalent)	Batch-controlled; authenticated by specialist	CER-AP rats	JAK2/STAT3/IL-17A signaling pathway	↓JAK2, ↓p-JAK2 (Tyr1007/1008), ↓STAT3, ↓p-STAT3 (Tyr705), ↓IL-17A	90

Note: All Latin binomials are derived from http://herb.ac.cn, with the exception of *Rheum palmatum L.* and *Salvia miltiorrhiza Bge.*^82^. Abbreviations: AKT, protein kinase B; AP, acute pancreatitis; CER, cerulein; DH-DS, Da Huang and Dan Shen; HPLC, high-performance liquid chromatography; HPLC-ESI-MS/UV, high-performance liquid chromatography-electrospray ionization-mass spectrometry/ultraviolet detector; IL, interleukin; JAK2, janus kinase 2; NaT, sodium taurocholate; NF-κB, nuclear factor kappa-B; NLRP3, NOD-, LRR- and pyrin domain-containing protein 3; PI3K, phosphoinositide 3-kinase; p-STAT, phosphorylated signal transduction and transcriptional activation factor; STAT, signal transducer and activator of transcription; Tyr, tyrosine; UHPLC-Orbitrap-MS/MS, ultra-high-performance liquid chromatography-Orbitrap tandem mass spectrometry; UHPLC-QE-MS, ultra-high-performance liquid chromatography-Q Exactive mass spectrometry; UHPLC-QTOF/MS, ultra-high-performance liquid chromatography-quadrupole time-of-flight mass spectrometry.

**Table 4:** Herbal-derived single compounds targeting the STATs pathway for AP treatment.

Compounds	Classification	Source	Preparation Method	Standardization	Cell/Animal Study	Mechanism	Function	Ref
Baicalein	Polyphenols	*Scutellaria baicalensis* Georgi (Huang Qin)	Normal saline solution (20 mg/kg, i.p.)	Purity > 98% (endotoxin-free); commercial source	CER-AP mice	↓p-p38, ↓p-ERK, ↓p-JNK, ↓p-JAK2, ↓p-STAT3	Inhibition of the MAPK/JAK2/STAT3 pathway alleviates pancreatic damage and reshapes the immune microenvironment	91
Baicalin	Polyphenols	*Scutellaria baicalensis* Georgi (Huang Qin)	Intravenous solution (20 mg/kg, i.v.)	Commercial source	ARG-AP HTG mice	↓B7H4, ↓p-JAK2, ↓p-JAK2 (Tyr1007/1008), ↓p-STAT3 (Tyr705)	Inhibition of the B7H4/JAK2/STAT3 axis mitigates oxidative stress and protects against pancreatic damage	92
Luteolin	Polyphenols	*Chrysanthemum indicum* L. (Ye Ju Hua)	DMSO-saline solution (2 mg/kg, i.p.)	Commercial source	CER-AP mice	↓p-STAT3 (Tyr705)	Targeting the STAT3 pathway to reduce pancreatic injury and inhibit inflammation-driven tumorigenesis	93
Daphnetin	Polyphenols	*Daphne odora* Thunb. (Rui Xiang)	5% DMSO solution (4 mg/kg, i.p.)	Purity 99.23%; commercial source	ARG-AP mice	↓p-JAK2, ↓p-JAK2 (Tyr1007/1008), ↓ p-STAT3 (Tyr705)	Inhibiting the JAK2/STAT3 pathway alleviates pancreatic and pulmonary injury while modulating immune responses	94
Honokiol	Polyphenols	*Magnolia officinalis* Rehd. et Wils. (Hou Po)	Saline solution (5 mg/kg, i.p.)	Commercial source	CER+LPS-AP mice	↓p-JAK1 (Tyr1034/1035), ↓p-JAK2 (Tyr1007/1008), ↓p-STAT1 (Ser727)	Targeting the JAK1/2-STAT1 pathway to alleviate pancreatic and intestinal tissue injury	95
Butyrate	Short-chain fatty acid	-	PBS solution (i.g. for 7 days)	Commercial source; GC-MS quantified	CER-AP mice	↓HDAC1, ↓p-STAT1 (Ser727), ↓NLRP3	Modulation of the HDAC1/STAT1/NLRP3 pathway to alleviate pancreatic and intestinal tissue injury	96
Curcumin	Polyphenols	*Curcuma longa* L. (Jiang Huang)	Saline solution (100 mg/kg, i.p.)	Commercial source	NaT-AP rats	↓p-JAK2, ↓p-STAT3 (Tyr705)	Inhibiting the JAK2/STAT3 pathway to alleviate pancreatic and renal tissue injury	97
DSC	Polyphenols	*Salvia miltiorrhiza* Bge. (Dan Shen)	Corn oil with <1% DMSO (25-100 mg/kg, i.p.)	Lab-synthesized; HPLC, 1H NMR, and ESI-HRMS identified	CER-AP 266-6 cells CER-AP mice	↓p-STAT3 (Tyr705), ↓NLRP3	Suppressing the STAT3/NLRP3 pathway to alleviate pancreatic injury and modulate immune responses	98
(R)-TML104	Polyphenols	*Veratrum album* L. (Bi Li Lu)	Saline solution (5, 10, 20 mg/kg, i.p.)	Lab-synthesized	CER-AP mice	↑SIRT1, ↓Ac-STAT3, ↓IL-6, ↓p-STAT3 (Tyr705)	Supressing the IL-6/STAT3 pathway to alleviate pancreatic injury	56
Selenium	Essential trace element	-	Saline solution (0.25-1 mg/kg, i.p.)	Commercial source	CER+LPS-AP mice	↓p-ERK, ↓p-JNK, ↓p-p38, ↓p-NF-κB, ↓p-STAT3	Targeting the MAPK/NF-κB/STAT3 pathway to attenuate pancreatic and pulmonary tissue injury	99
Picroside II	Terpenoids	*Neopicrorhiza scrophulariiflora* (Pennell) Hong (Hu Huang Lian)	Saline solution (25 mg/kg, i.p.)	Purity > 98%; commercial source	NaT-AP rats	↓p-JAK2, ↓p-STAT3	Inhibition of the JAK2/STAT3 pathway to alleviate pancreatic and hepatic tissue injury	100
Limonin	Terpenoids	*Citrus limon* (L.) Burm. f. (Ning Meng)	Tail vein injection (25, 50, 100 mg/kg, i.v.)	Commercial source	CER-AP mice ARG-AP mice	↓JAK2, ↓p-STAT3 (Tyr705)	Regulation of the JAK2/STAT3 pathway to alleviate pancreatic injury in both MAP and SAP model	101
Lutein	Terpenoids	Green leafy vegetables, fruits, and egg yolk	DMSO solution (1-5 μM)	Commercial source	CER-AP AR42J cells	↑PPAR-γ, ↑SOCS3, ↓p-JAK2 (Tyr1007/1008), ↓p-STAT3 (Tyr705)	Targeting the PPAR-γ/SOCS3/JAK2/STAT3 pathway to alleviate pancreatic injury	102
Escin Sodium	Terpenoids	*Aesculus chinensis* Bunge (Qi Ye Shu)	Saline solution (1-6 mg/kg, i.p.)	Commercial source	NaT-AP AR42J cells NaT-AP rats	↓p-ERK, ↓p-STAT3 (Tyr705)	Suppression of the ERK/STAT3 pathway to alleviate pancreatic injury	103
Glycyrrhizin	Terpenoids	*Glycyrrhiza uralensis* Fisch. (Gan Cao)	-	Commercial source	NaT-AP PACs NaT-AP mice	↓p-ERK1/2, ↓p-STAT3, ↓p-AKT	Modulation of the MAPK/ERK/STAT3/AKT pathway to mitigate pancreatic injury	104
Rhein	Anthraquinones	*Rheum palmatum* L. (Da Huang)	Saline solution (30 mg/kg, i.g.)	Purity > 98%; commercial source	CER-AP AR42J cells NaT-AP rats	↓p-JAK2 (Tyr1007/1008), ↓JAK2; ↓p-STAT3 (Tyr705), ↓STAT3	Inhibition of the JAK2/STAT3 pathway attenuates pancreatic damage	81
Colchicine	Alkaloids	*Colchicum autumnale* L. (Qiu Shui Xian)	Saline solution (0.5 mg/kg, i.g.)	Commercial source	NaT-AP rats	↓p-STAT3 (Tyr705)	Suppression of STAT3 signaling alleviates pancreatic and pulmonary tissue injury	107
Rutaecarpine	Alkaloids	*Euodia rutaecarpa* (Juss.) Benth. (Wu Zhu Yu)	DMSO solution (25, 50, 100 mg/kg, i.g.)	Purity > 98%; commercial source	CER+LPS-AP mice CER-AP AR42J cells	↓p-p38, ↓p-ERK, ↓p-JNK, ↓p-STAT3 (Ser727)	Modulation of the MAPK/STAT3 pathway to mitigate pancreatic injury	108
Kinsenoside	Glycosides	*Anoectochilus roxburghii* (Wall.) Lindl. (Jin Xian Lian)	Saline solution (2.5, 5, 10 mg/kg, i.p.)	Purity 99.91%; Commercial source	CER+LPS-AP mice NaT-AP mice	↓p-STAT1 (Tyr701)	Targeting the STAT1 pathway alleviates pancreatic injury while modulating immune responses	109
Lactate	Metabolic byproduct	Endogenous metabolite	PBS solution (150 mM, i.p.)	Commercial source	CER-AP PACs CER-AP mice	↓p-JAK2 (Tyr1007/1008), ↓p-STAT1 (Tyr701)	Inhibiting the JAK2/STAT1 pathway alleviates pancreatic injury while modulating immune responses	110

Note: All Latin names are from http://herb.ac.cn, except those marked with an asterisk (*), which follow the 2020 Pharmacopoeia of the People's Republic of China. Abbreviations: Ac, acetylated; AKT, protein kinase B; AP, acute pancreatitis; ARG, arginine; B7H4, B7 homolog 4; CER, cerulein; DSC, 4-(2-acetoxy-3-((R)-3-(benzylthio)-1-methoxy-1-oxopropan-2-ylamino)-3-oxopropyl)-1,2-phenylene diacetate; EGFR, epidermal growth factor receptor; ERK, extracellular signal-regulated kinase; HDAC1, histone deacetylase 1; HTG, hypertriglyceridemia; i.g., intragastric; i.p., intraperitoneal; i.v., intravenous; JAK, janus kinase; JNK, c-Jun N-terminal kinase; LPS, lipopolysaccharide; MAP, mild acute pancreatitis; MAPK, mitogen-activated protein kinase; NaT, sodium taurocholate; NF-κB, nuclear factor kappa-B; NLRP3, NOD-, LRR- and Pyrin domain-containing protein 3; PACs, pancreatic acinar cells; PPAR-γ, peroxisome proliferator-activated receptor gamma; p-STAT, phosphorylated signal transduction and transcriptional activation factor; (R)-TML104, (R)-4,6-dimethoxy-3-(4-methoxy phenyl)-2,3-dihydro-1H-indanone; SAP: severe acute pancreatitis; Ser, serine; SIRT1, sirtuin 1; SOCS3, suppressor of cytokine signaling 3; SOX9, SRY-box transcription factor 9; STAT, signal transducer and activator of transcription; Tyr, tyrosine.

## Abbreviations

ADM: acinar-to-ductal metaplasia
AKT: protein kinase B
AP: acute pancreatitis
ARG: arginine
BISAP: Bedside Index of Severity in Acute Pancreatitis
CER: cerulein
CQCQD: Chaiqin Chengqi Decoction
DCQD: Da Cheng Qi Decoction
DSC: 4-(2-acetoxy-3-((R)-3-(benzylthio)-1-methoxy-1-oxopropan-2-ylamino)-3-oxopropyl)-1:2-phenylene diacetate
DH-DS: Dahuang and Danshen
DEX: dexamethasone
DHFZT: Da-Huang-Fu-Zi-Tang
ERK: extracellular signal-regulated kinase
HIP: Hepatocarcinoma-intestine-pancreas
HPLC: high-performance liquid chromatography
HMGB1: high-mobility group protein B1
IFN-γ: interferon gamma
IL: interleukin
JAK: janus kinase
MAP: mild acute pancreatitis
MAPK: mitogen-activated protein kinase
MDA: malondialdehyde
MODS: multiple organ dysfunction syndrome
MPO: myeloperoxidase
NaT: sodium taurocholate
NF-κB: nuclear factor kappa-B
NLRP3: nucleotide-binding oligomerization domain like receptor (NLR) family pyrin domain containing 3
OD: organ dysfunction
PACs: pancreatic acinar cells
PAP: pancreatitis-associated protein
P-gp: P-glycoprotein
PI3K: phosphoinositide 3-kinase
PK: pharmacokinetic
PPAR-γ: peroxisome proliferator-activated receptor gamma
PSCs: pancreatic stellate cells
QYG: Qingyi Granules
(R)-TML104: (R)-4:6-dimethoxy-3-(4-methoxy phenyl)-2:3-dihydro-1H-indanone
SAP: severe acute pancreatitis
SIRS: systemic inflammatory response syndrome
SIRT1: sirtuin 1
SMI: Shenmai injection
SOCS: suppressor of cytokine signaling
STAT: signal transducer and activator of transcription
TCM: traditional chinese medicine
THTR: thiamine transporter
TLR4: toll-like receptor 4
TNF-α: tumor necrosis factor alpha
VB1: vitamin B1
